# Multifaceted Roles of Ferroptosis in Lung Diseases

**DOI:** 10.3389/fmolb.2022.919187

**Published:** 2022-06-24

**Authors:** Yi Li, Ying Yang, Yongfeng Yang

**Affiliations:** ^1^ Institute of Respiratory Health, West China Hospital, Sichuan University, Chengdu, China; ^2^ Precision Medicine Key Laboratory, West China Hospital, Sichuan University, Chengdu, China; ^3^ Department of Respiratory and Critical Care Medicine, West China Hospital, Sichuan University, Chengdu, China

**Keywords:** ferroptosis, lung disease, infection, injury, cancer

## Abstract

Ferroptosis is a distinct type of programmed cell death (PCD) that depends on iron and is characterized by the accumulation of intracellular iron, exhaustion of glutathione, deactivation of glutathione peroxidase, and promotion of lipid peroxidation. Recently, accumulated investigations have demonstrated that ferroptosis is strongly correlated with the initiation and development of many lung diseases. In this review, we summarized the contribution of ferroptosis to the pathologic process of lung diseases, namely, obstructive lung diseases (chronic obstructive pulmonary disease, asthma, and cystic fibrosis), interstitial lung diseases (pulmonary fibrosis of different causes), pulmonary diseases of vascular origin (ischemia-reperfusion injury and pulmonary hypertension), pulmonary infections (bacteria, viruses, and fungi), acute lung injury, acute respiratory distress syndrome, obstructive sleep apnea, pulmonary alveolar proteinosis, and lung cancer. We also discussed the therapeutic potential of targeting ferroptosis for these lung diseases.

## Introduction

**FIGURE 1:**
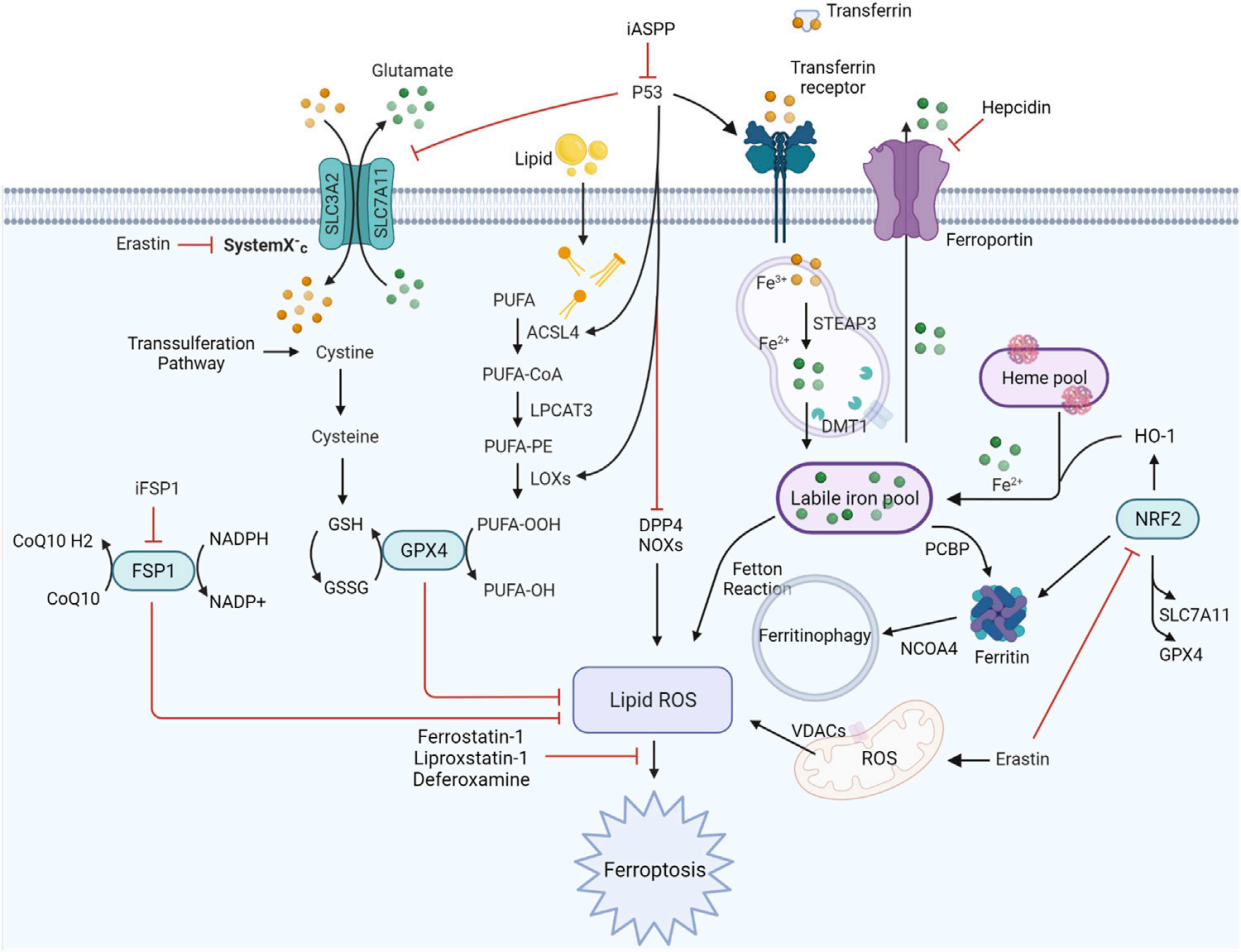
Regulatory signaling pathways implicated in ferroptosis. The features of ferroptosis include the substrate of lipid peroxidation (PUFA), executor of lipid peroxidation (iron metabolism), and anti-ferroptosis systems (GPX4-centered and p53-centered systems). ACSL4, acyl-CoA synthetase long-chain family member four; DDP4, dipeptidyl peptidase-4; DMT1, divalent metal transporter 1; FSP1, ferroptosis suppressor protein 1; GPX4, glutathione peroxidase four; GSH, glutathione; HO-1, heme oxygenase 1; iASPP, inhibitor of apoptosis stimulating protein of p53; iFSP1, FSP1 inhibitor 1; LOXs, lipoxygenases; LPCAT3, lysophosphatidylcholine acyltransferase three; NRF2, nuclear factor erythroid 2-related factor 2; NCOA4, nuclear receptor coactivator four; PCBP, poly (RC)-binding proteins; PUFA, polyunsaturated fatty acid; ROS, reactive oxygen species; SLC3A2, solute carrier family three member 2; SLC7A11, solute carrier family seven member 11; STEAP3, six-transmembrane epithelial antigen of prostate three; VDACs, voltage-dependent anion channels.

**FIGURE 2:**
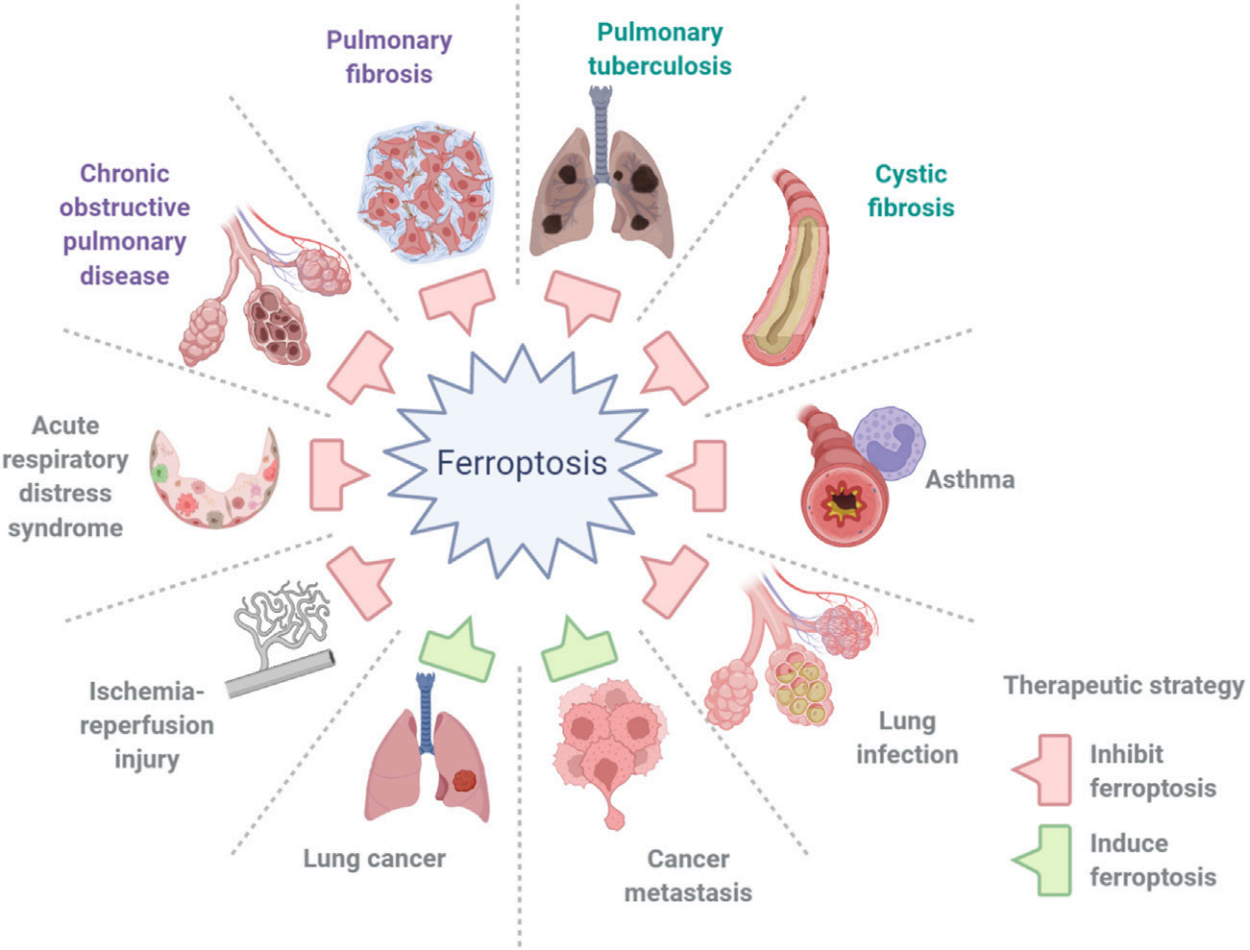
Ferroptosis in lung diseases. Ferroptosis contributes greatly to the pathogenesis of lung diseases, including chronic obstructive pulmonary disease, asthma, cystic fibrosis, pulmonary fibrosis, ischemia–reperfusion injury, pulmonary infections, acute lung injury and acute respiratory distress syndrome, and lung cancer and metastasis.

The term “ferroptosis” was first coined by Dixon et al. (2012)to define a previously unknown form of programmed cell death (PCD) elicited by erastin in Ras-mutant cells in 2012. It is dependent on iron and is characterized by the accumulation of intracellular iron, exhaustion of glutathione, deactivation of glutathione peroxidase, and promotion of lipid peroxidation (Figure 1). Iron can catalyze the formation of free radicals from reactive oxygen species (ROS) *via* the Fenton reaction, which is the reduction of H_2_O_2_ by a single electron to produce a hydroxyl radical. Ferroptosis is distinct from other types of cell death, such as apoptosis, autophagy, pyroptosis, or necrosis, in morphology, genetics, metabolism, and molecular biology. The specific morphology of ferroptosis includes intact cytomembranes, cellular shrinkage, enhanced mitochondrial membrane density, reduced or absent mitochondrial cristae, crumpling of the mitochondrial membrane, rupture of the outer membrane, and regular nucleus size without concentrated chromatin (Dixon et al., 2012; Yang and Stockwell, 2016). However, apoptosis is characterized by typical apoptotic cellular bodies without rupture of cell membranes, while necrosis is characterized by the occurrence of cell swelling, nucleus concentration, fragmentation and dissolution, chromatin staining and flocculence, and organelle enlargement or fragmentation. In terms of pathological processes, cell apoptosis increases ROS, Ca^2+^, and pH levels, which activates cysteinyl aspartate-specific proteinase and releases cytochrome c to promote caspase-3/7. During cell necrosis, the inflammatory response is induced by the activation of several signaling pathways, namely, Toll-like receptor 4/myeloid differentiation primary response gene 88 (TLR4/Myd88)-dependent tumor necrosis factor (TNF) and receptor-interacting protein kinases (RIPK). Ferroptosis is regulated by glutathione peroxidase 4 (GPX4), which directly converts lipid hydroperoxides (L‐OOH) to nontoxic lipid alcohols (L‐OH) (Yang et al., 2014; Forcina and Dixon, 2019; and Stockwell et al., 2020). Later, a shred of evidence showed that p53 influences ferroptosis, it was reported and demonstrated that p53 is a key regulator of both classical ferroptosis pathways and noncanonical ferroptosis pathways, such as arachidonate 12-lipoxygenase (ALOX12) and iPLA2β. (Jiang et al., 2015a; Liu and Gu, 2021; Liu and Gu, 2022). Besides, ferroptosis is manipulated by several signaling pathways, such as p62-kelch-like ECH-associated protein (Keap1)-nuclear factor erythroid 2-related factor 2 (Nrf2)/heme oxygenase-1 (HO-1) (Sun et al., 2016; Dodson et al., 2019) and acyl-CoA synthetase long-chain family member 4 (ACSL4) (Doll et al., 2017). In addition, ferroptosis can be prevented by iron chelation or by the use of a lipophilic antioxidant but not by inhibitors of other forms of cell death. Aberrant regulation of ferroptosis has been implicated in disease pathogenesis in the heart, kidney, brain, liver, and lung (Zheng and Conrad, 2020). The contributions of ferroptosis to the pathologic process of multiple lung diseases have been recognized. In addition, many interventions, including ferroptosis inducers and inhibitors, iron chelators, lipid peroxidation inhibitors, and antioxidants, have been introduced into ferroptosis-related lung diseases (Table 1). In this review, we will summarize the recent understanding of ferroptosis in lung diseases and provide a new angle for future research on the pathologic process and clinical treatment of lung diseases (Figure 2).

**TABLE 1:** Reagents and mechanisms of ferroptosis in lung diseases.

Disease	Reagent	Proposed mechanism	References
COPD	Deferoxamine	Chelate iron and block iron-dependent lipid peroxidation	Ghio et al. (2008a); Yoshida et al. (2019); Tang et al. (2021a)
	Ferrostatin-1	Inhibit lipid peroxidation	Yoshida et al. (2019); Tang et al. (2021a); Lian et al. (2021)
	NAC	Recover intracellular cysteine	
	Curcumin	Activate GPX4; Upregulate SLC7A11	Tang et al. (2021a)
	Dihydroquercetin	Regulate NRF2	Liu et al. (2022a)
Asthma	Deferoxamine	Chelate iron and block iron-dependent lipid peroxidation	Wenzel et al. (2017)
	Ferrostatin-1	Inhibit lipid peroxidation	Wenzel et al. (2017); Yang and Shang, (2022)
	3-methyladenine	Upregulate system Xc-; Activate GPX4	Yang and Shang, (2022)
	Acupuncture	Downregulate SLC3A2 and ATP1A3	Tang et al. (2021c)
	Erastin	Block cysteine import, inducing GSH depletion and GPX4 inactivation	Wu et al. (2020)
	RSL3	Covalently inhibit GPX4, causing accumulation of lipid peroxidation	Wu et al. (2020)
	Artesunate	Activate ferritinophage to increases iron abundance	Wu et al. (2020)
Cystic fibrosis	Deferoxamine	Chelate iron and block iron-dependent lipid peroxidation	Maniam et al. (2021)
	Ferrostatin-1	Inhibit lipid peroxidation	Maniam et al. (2021)
Pulmonary fibrosis	Deferoxamine	Chelate iron and block iron-dependent lipid peroxidation	(Cheng et al., 2021; Takahashi et al., 2021)
	Liproxstatin-1	Inhibit lipid peroxidation	He et al. (2021)
	Phytic acid	Chelate iron and block iron-dependent lipid peroxidation	
Ischemia–Reperfusion Injury	Liproxstatin-1	Inhibit lipid peroxidation	Xu et al. (2020)
	Rosiglitazone	Inhibition of ACSL4	Xu et al. (2020)
	Irisin	Regulate Nrf2/HO-1 axis; activates GPX4; inhibition of ACSL4	Wang et al. (2022a)
	Lidocaine	Regulate the p38/MAPK; increase FTH1 and GPX4; decrease Tf	Ma et al. (2022)
	Deferoxamine	Chelate iron and block iron-dependent lipid peroxidation	Lui et al. (2013)
	Pirfenidone	Chelate iron and block iron-dependent lipid peroxidation	Liu et al. (2005)
Pulmonary hypertension	Ferrostatin-1	Inhibit lipid peroxidation	Xie et al. (2022)
	Deferoxamine	Chelate iron and block iron-dependent lipid peroxidation	Jasenosky et al. (2015)
Infection: *P. aeruginosa*	Ferrostatin-1	Inhibit lipid peroxidation	Ousingsawat et al. (2021)
	Baicalein	Suppress peroxidation of polyunsaturated fatty acids	Dar et al. (2022)
	CoQ10	Inhibit lipid peroxidation	Ousingsawat et al. (2021)
	Idebenone	Inhibit lipid peroxidation	Ousingsawat et al. (2021)
Pulmonary tuberculosis	Ferrostatin-1	Inhibit lipid peroxidation	Amaral et al. (2019)
	Glutathione	Recover intracellular cysteine	Pan et al. (2020)
	Vitamin E	Inhibit lipid peroxidation	Seyedrezazadeh et al. (2008)
Infection: Gram-negative bacteria	Liproxstatin-1	Inhibit lipid peroxidation	Klobucar et al. (2021)
COVID-19	Vitamin C	Inhibit lipid peroxidation	Chavarría et al. (2021)
	Vitamin E	Inhibit lipid peroxidation	Chavarría et al. (2021)
	NAC	Recover intracellular cysteine	Jaiswal et al. (2020); Chavarría et al. (2021)
	Melatonin	Chelate iron and block iron-dependent lipid peroxidation	Chavarría et al. (2021)
	Deferoxamine	Chelate iron; inhibit viral replication; immunomodulation; downregulate hepcidin I	Dalamaga et al. (2020)
	Deferiprone	Chelate iron; inhibit viral replication; immunomodulation	Dalamaga et al. (2020)
	Deferasirox	Chelate iron; inhibit viral replication; immunomodulation	Dalamaga et al. (2020)
	Lactoferrin	Bind iron and inhibit viral replication	Carota et al. (2021)
	Methemoglobin reductase	Chelate iron and	Muhoberac, (2020)
	Glutathione	Recover intracellular cysteine	Silvagno et al. (2020)
Infection:Zygomycetes	Melatonin	Chelate iron; inhibit microbial activity	Sen, (2021)
	Deferoxamine	Chelate iron and block iron-dependent lipid peroxidation	Kontoghiorghes et al. (2010); Álvarez et al. (2013)
	Deferiprone	Chelate iron; inhibit microbial activity with better tissue penetration capacity	Kontoghiorghes et al. (2010); Álvarez et al. (2013)
	Deferirox	Chelate iron; inhibit microbial activity	Kontoghiorghes et al. (2010); Álvarez et al. (2013)
Infection: A. Fumigatus	Deferoxamine	Chelate iron and block iron-dependent lipid peroxidation	Hsu et al. (2018)
	Deferiprone	Chelate iron and block iron-dependent lipid peroxidation	Hsu et al. (2018)
	Deferirox	Chelate iron and block iron-dependent lipid peroxidation	Hsu et al. (2018)
Acute lung injury/acute respiratory distress syndrome	iASPP	Regulate p53 and NRF2	Li et al. (2020a)
	Ferrostatin-1	Inhibit lipid peroxidation	Liu et al. (2020)
	Liproxstatin-1	Inhibit lipid peroxidation	Li et al. (2020a)
	Panaxadiol	Regulate KEAP1/NRF2/HO-1 axis; activates GSH and GPX4	Li et al. (2021b)
	4-octyl itaconate	Regulate NRF2/HO-1 axis; activates GSH and GPX4	He et al. (2022b)
	Obacunone	Regulate NRF2/HO-1 axis; activates GSH and GPX4	Li et al. (2022)
	Puerarin	Activate SLC7A11, GPX4, and FTH1; inhibit NOX1 to reduce lipid ROS generation	Xu et al. (2021a)
	Acupuncture	Activate SLC7A11, GPX4, and FTH1	Zhang et al. (2022)
Lung cancer/metastasis	Erastin	Block cysteine import, inducing GSH depletion and GPX4 inactivation	Huang et al. (2018); Pan et al. (2019)
	RSL3	Covalently inhibit GPX4, causing accumulation of lipid peroxidation	Zhang et al. (2020b); Liu et al. (2021b)
	Cisplatin	Block cysteine import, inducing GSH depletion and GPX4 inactivation	Guo et al. (2018)
	PRLX93936	Regulate NRF2/HO-1 axis; GSH depletion and GPX4 inactivation	Liang et al. (2021)
	Imidazole ketone erastin	Block cysteine import, inducing GSH depletion and GPX4 inactivation	Ye et al. (2020)
	Sorafenib	Block cysteine import, inducing GSH depletion and GPX4 inactivation	Li et al. (2020b)
	Levobupivacaine	Increases iron abundance and mediate iron-dependent lipid peroxidation	Meng et al. (2021)
	BEBT-908	Regulate p53 and STAT1	Fan et al. (2021)
	iFSP1	Inhibit FSP1–CoQ10–NAD(P)H system	Doll et al. (2019)
	Erianin	Induce GSH depletion and mediate iron-dependent lipid peroxidation	Chen et al. (2020b)
	Sanguinarine	Inhibit GSH and GPX4	Xu et al. (2022)
	Dihydroisotanshinone I	Inhibit GSH and GPX4	Wu et al. (2021)

### Chronic Obstructive Pulmonary Disease

At present, chronic obstructive pulmonary disease (COPD) is the fourth leading cause of morbidity and mortality worldwide and is still increasing (Barnes et al., 2015; Barnes et al., 2019). It is characterized by chronic airway inflammation, lung destruction, and remodeling, resulting in irreversible airflow obstruction. Cigarette smoke (CS) exposure is the main risk factor for COPD due to its high concentration of ROS. The consequent cellular oxidative stress provokes inflammation, cell senescence, and death. Early studies have demonstrated that accumulated iron and ferritin and increased serum ferritin and nonheme iron were observed in lung epithelial cells and alveolar macrophages during exposure to CS (Wesselius et al., 1994; Ghio et al., 2008a). Mitochondrial dysfunction and endoplasmic reticulum stress are usually observed in the cytoplasm, and ferroptosis occurs in bronchial epithelial cells (Park et al., 2019). The increased ferritinophagy mediated by nuclear receptor coactivator 4 (NCOA4) and reduction of glutathione peroxidase 4 (GPX4) led to the accumulation of free iron and lipid peroxidation during CS exposure. Moreover, GPX4^+/−^mice showed significantly higher degrees of lipid peroxidation and an enhanced COPD phenotype than wild-type mice, whereas these phenotypes could be attenuated in GPX-transgenic mice (Dowdle et al., 2014; Yoshida et al., 2019). PM2.5 is another risk factor for COPD. Increased cellular iron content and ROS production in human endothelial cells were observed after inhaling PM2.5 particles, while the levels of glutathione (GSH) and nicotinamide adenine dinucleotide phosphate (NADPH) decreased. Iron overload and redox imbalance caused by TFRC and ferritin dysregulation are the major inducers of ferroptosis (Wang and Tang, 2019). The abovementioned investigations indicated that ferroptosis is involved and plays a crucial damaging role in COPD (Mizumura and Gon, 2021), and searching for an accurate inhibitor of ferroptosis to delay the progression and prevent the occurrence of COPD is pivotal to the forthcoming research. Experimental interventions, such as the iron chelator deferoxamine, the ferroptosis inhibitor ferrostatin-1, and suppression of lipid peroxidation by GPX4, could effectively reduce lipid peroxidation, upregulate GSH and NADPH levels, and inhibit ferroptosis (Ghio et al., 2008a; Yoshida et al., 2019; Tang et al., 2021a; and Lian et al., 2021). Moreover, recent reports revealed that antioxidants, such as N-acetyl-l-cysteine (NAC) and curcumin, could improve the reduction of GSH and reduce lipid peroxidation (Tang et al., 2021a), while dihydroquercetin could inhibit ferroptosis in lung epithelial cells by activating the Nrf2-mediated pathway (Liu et al., 2022a).

### Asthma

Asthma is a chronic inflammatory respiratory disorder that results in intermittent episodes of wheezing, breathlessness, chest stuffiness, and cough. The distinguishing features of the disease are sporadic and reversible airway obstruction, chronic bronchial inflammation, bronchial smooth muscle cell hypertrophy and hyperreactivity, and increased mucus secretion (Papi et al., 2018). The function of type-2 helper T (Th2) cells is critical to the pathologic process of asthma. Upon activation, Th2 cells produce cytokines and active mediators, such as interleukin-4 (IL-4), which stimulate IgE secretion by B cells, IL-5 triggers eosinophils, and IL-13 arouses mucus and IgE production. Meanwhile, mast cells, macrophages, neutrophils, and T cells are activated and gather in the airways and secrete inflammatory mediators, resulting in chronic airway inflammation (Hammad and Lambrecht, 2021). Continuous studies have demonstrated that airway epithelial cells from asthmatic patients actively produced 15-lipoxygenase-1 (15-LOX1) and 15-hydroxyeicosatetraenoic acid (15-HETE). The reports demonstrated that 15LOX1-phosphatidylethanolamine-binding protein 1 (PEBP1) complex activation consistently stimulates IL-13/IL-4-mediated Th2 inflammation, including mucin 5AC (MUC5AC), periostin (POSTN), and chemokine (C-C motif) ligand 26 (CCL26). 15-LOX1-PEBP1 can activate autophagy and ferroptosis. Besides, there was a positive correlation between the colocalization of 15LOX1/PEBP1 puncta in freshly brushed airway epithelial cells and the fraction of exhaled nitric oxide (FeNO) in asthmatic patients. The ferroptosis inducer Ras-selective lethal small molecule 3 (RSL3) significantly promotes the occurrence of lipid peroxidation and ferroptosis in IL-13-treated human airway epithelial cells (Zhao et al., 2009; Zhao et al., 2011; Wenzel et al., 2017; Zhao et al., 2020a; Nagasaki et al., 2022). In an experimental model of house dust mite-induced asthma, an association between elevated lung iron levels in airway tissue, increased ROS and lipid peroxidation and decreased GSH levels in the lungs was observed (Tang et al., 2021b), suggesting the involvement of ferroptosis in the pathogenesis of allergic asthma. These results reveal the correlation between ferroptosis and the pathobiologic pathway of asthma and the possibility of targeted inhibition of ferroptosis. Ferrostatin-1 and deferoxamine are introduced to reduce sensitivity to ferroptosis (Wenzel et al., 2017; Yang and Shang, 2022); otherwise, the 15LOX1/PEBP1 pathway is under investigation as a novel asthma therapeutic target to regulate the ferroptotic cell death (Zhao et al., 2011). On the other hand, 3-methyladenine was proven to ameliorate ferroptosis in an ovalbumin (OVA)-induced asthma model by suppressing the production of ROS and inflammatory cytokines, while upregulating superoxide dismutase (SOD) (Yang and Shang, 2022). In addition, acupuncture was reported to be effective in treating asthma by reducing solute carrier family 3 member 2 (SLC3A2) and ATPase Na+/K+ transporting subunit alpha 3 (ATP1A3) expression, oxidative stress, and inflammatory cytokine levels and was recently connected with the manipulation of ferroptosis (Tang et al., 2021c).

Eosinophils are vital in allergic disorders, such as asthma, and prompting eosinophil death, which effectively weakens inflammation (Hammad and Lambrecht, 2021). The increased number and prolonged survival time of eosinophils in asthma suggest the selected targeting of ferroptosis as a promising therapeutic strategy for airway inflammation. Ferroptosis-inducing agents, namely, erastin, RSL3, and artesunate (ART), were proven to induce eosinophil ferroptosis and collaborate with dexamethasone to promote eosinophil death. Targeted administration of eosinophil ferroptosis appeared to relieve allergic airway inflammation and might cut the dosage and reduce the side effects of glucocorticoids in an OVA-induced asthma model (Wu et al., 2020). The concept of inducing ferroptosis of specific immune cells to diminish airway inflammation and alleviate mediator production is promising but needs to be investigated for its clinical potential in future studies of asthma.

### Cystic Fibrosis

Cystic fibrosis (CF) is an autosomal recessive genetic disease that causes dysfunction of ion transport in the exocrine glands (Ratjen and Döring, 2003). It is the most common fatal genetic disorder among white populations. The disease is due to defects in the cystic fibrosis transmembrane conductance regulator (CFTR) gene, resulting in abnormalities in chloride transport in epithelial transport affecting fluid secretion in exocrine glands and respiratory, gastrointestinal, and reproductive tracts. Recurrent respiratory infection and pancreatic dysfunction are the two most important clinical symptoms (Prentice et al., 2021). In patients with CF, the excretion of chloride ions in the airway is reduced and the resorption of sodium and water is increased, which results in dehydration of mucous layer coating epithelial cells, loss of mucociliary function, mucus blockage, and mucous plugging. A recent report demonstrated that CF airway epithelial cells exposed to ferric ammonium citrate (FAC) and the ferroptosis inducer erastin are susceptible to ferroptosis compared to isogenic CFTR-corrected airway epithelial cells *in vitro* (Maniam et al., 2021). Ferrostatin-1 and deferoxamine are able to inhibit ferroptosis by alleviating iron accumulation and lipid peroxidation and thus, may be potential therapeutic interventions for CF. On the other hand, patients with CF commonly develop respiratory infections caused by *Pseudomonas aeruginosa*, *Staphylococcus aureus*, *Hemophilus influenzae*, and *Burkholderia cepacia*. Among these, *P. aeruginosa* is an important opportunistic pathogen responsible for the ferroptotic cell death in airways, as discussed later in the section on pulmonary infections.

### Obstructive Sleep Apnea

Obstructive sleep apnea (OSA) is a respiratory disease characterized by an intermittent nocturnal decrease in oxygenated hemoglobin and sleep interruption (Patel et al., 2019). The main feature of OSA is chronic intermittent hypoxia (CIH) from the view of pathophysiology. Cardiovascular diseases, liver diseases, and metabolic diseases are the major health risks to OSA patients. Although there is no report of ferroptosis caused by hypoxia-reperfusion in lung diseases, oxidative stress, and lipid peroxidation have been reported to induce liver injury in OSA (Chen et al., 2020a; Chen et al., 2021a). Both reports revealed that ferroptosis mediated liver injury induced by CIH in rodent models, as evidenced by the increase in lipid peroxidation, the decrease in GPX4 expression, and the increase in ACSL4 expression.

### Pulmonary Fibrosis

Idiopathic pulmonary fibrosis (IPF) refers to a refractory and irreversible progressive pulmonary fibrotic disorder without clear etiology. The characteristic feature of IPF is patchy but progressive bilateral interstitial fibrosis, leading to severe hypoxemia and cyanosis in advanced cases (Lederer et al., 2018). Recurrent epithelial activation and injury is the leading cause of IPF occurrence. Epithelial repair deficiency and inflammation at damage sites give rise to the vigorous proliferation of fibroblasts and myofibroblasts, resulting in typical fibroblastic foci. Ferroptosis-related genes, which includes neuroblastoma RAS viral (v-ras) oncogene homolog (NRAS) and MUC1, but not limited to them, were elevated in bronchoalveolar lavage fluid and cells in IPF patients, which suggests the participation of ferroptosis in the disease and may be used for prognostic prediction of IPF (Li et al., 2021a; He et al., 2021; He et al., 2022a).

Pulmonary fibrosis (PF) is an interstitial lung disease that derives from long-term inhalation of CS and dust, such as asbestos, silica, and coal, the use of drugs, such as bleomycin and amiodarone, accidental exposure to paraquat, or lung injury are caused by radiation therapy. A previous study showed that erastin promoted transforming growth factor β1 (TGF-β1)-induced fibroblast-to-myofibroblast differentiation by promoting ROS and lipid peroxidation and hindering GPX4 expression, resulting in collagen accumulation and destruction of the alveolar structure, while ferrostatin-1 may inhibit this process (Gong et al., 2019). A recent report demonstrated that the upregulated long noncoding RNA (lncRNA) zinc finger antisense 1 (ZFAS1) is positively correlated with SLC38A1 expression in bleomycin-induced PF rat lung tissue and in TGF-β1-induced human fetal lung fibroblast cells. Experiments have shown that inhibition by knockdown or silencing of the lncRNA ZFAS1 can significantly attenuate lipid peroxidation and inflammation, thus, inhibiting ferroptosis and progression of PF induced by bleomycin (Yang et al., 2020). Another study demonstrated that SET domain bifurcated 1 (SETDB1) and H3K9me3 expression was downregulated in a bleomycin-induced PF rat model, leading to the induction of epithelial-mesenchymal transition and increased lipid ROS, ferrous ions, and ferroptosis (Liu et al., 2022b). On the other hand, inducing ferroptosis by erastin could elicit iron accumulation, ROS production, epithelial-mesenchymal transition, and autophagy in lung epithelial cell lines, as evidenced by upregulated microtubule-associated protein 1A/1B-light chain 3 (MAP1LC3) and Beclin 1 (BECN1) (Han et al., 2021; Sun et al., 2021). Links between pulmonary fibrosis and ferroptosis are complicated and still under investigation. Experimental interventions, such as deferoxamine, effectively reduced iron accumulation and lipid peroxidation, thus, inhibiting ferroptosis in a bleomycin-induced PF cell and mouse model (Cheng et al., 2021; Takahashi et al., 2021).

Asbestos is a silicate mineral containing iron, magnesium, and calcium with a core of SiO_2_. It has been established that asbestos fibers tend to accumulate near the mesothelial cell layer, where they produce ROS, leading to DNA damage and potential carcinogenic mutations. Workers exposed to asbestos can develop lung cancer and malignant mesothelioma. Iron homeostasis is changed among patients with asbestosis, as evidenced by the accumulation of iron, ferritin, divalent metal transporter 1 (DMT1), and ferroportin 1 (FPN1) in the lung autopsy. Iron chelators, including deferoxamine and phytic acid, were utilized in asbestos- and silica-induced PF animal models, which shows that procollagen and inflammation could partly reversed, whereas glutathione had no effect.

Paraquat (PQ) is an economic and effective herbicide that could cause multiple organ acute injury, lung fibrosis, multiple organ failure, and death due to unintentional or intentional inhalation, ingestion, and percutaneous absorption. Evidence of the connection between PQ poisoning and ferroptosis is emerging, confirmed by the formation of high-energy oxygen free radicals and lipid peroxidation (Rashidipour et al., 2020). The hypothesis that ferroptosis inhibitors might be introduced in PQ-induced PF was raised and needs to be confirmed.

Radiation-induced lung fibrosis (RILF) is a life-threatening complication after radiotherapy of chest tumors. Collagen deposition, decreased GPX4 expression, and increased ROS were observed in the mouse model, indicating that ferroptosis was involved when exposed to radiation. The ferroptosis inhibitor liproxstatin-1 alleviated RILF by activating the Nrf2 pathway in this experimental study (Li et al., 2019). Therefore, strategies for regulating iron metabolism and controlling ferroptosis can be exploited to delay the progression of PF in future experimental and clinical practice.

### Lung Ischemia-Reperfusion Injury

An ischemia injury arises at the initial stage of vascular compromise, however, airway epithelial cells are relatively resistant to transient hypoxia. Reperfusion injury is caused by recovery of blood flow and is related to overwhelming damage. Multiple organ failure might arise in serious cases. Although the underlying mechanisms of reperfusion injury are not fully understood, they involve the production of free radicals, neutrophil infiltration, and the secretion of inflammatory mediators, namely, cytokines, chemokines, and complements. Clinical disorders associated with the development of ischemia-reperfusion include pulmonary thromboembolism and thrombolysis, lung resection and trauma, lung transplantation, and other operations. Increased iron content and lipid peroxidation accumulation, together with decreased GPX4 and elevated ACSL4 expression, were detected in ischemia-reperfusion lungs. Treatment with liproxstatin-1 to inhibit ferroptosis, administration of the ACSL4 inhibitor rosiglitazone before ischemia, and ACSL4 knockdown ameliorated lung ischemia-reperfusion injury by protecting against ferroptotic damage in animal and cell models (Xu et al., 2020). Irisin, a novel muscle-derived myokine, was reported to suppress ferroptosis in lung ischemia-reperfusion damage *in vitro* and *vivo*, as confirmed by lower ROS, malondialdehyde (MDA), and iron accumulation, along with alterations in GPX4 and ACSL4 (Wang et al., 2022a). Lidocaine attenuated inflammation, apoptosis, and ferroptosis in a lung epithelial cell line by controlling the p38/mitogen-activated protein kinase (MAPK) pathway (Ma et al., 2022).

Furthermore, there were elevated iron concentrations in alveolar fluid and tissue in human lung allografts (Baz et al., 1997), which could increase the risk of lung allografts to iron radicals, ROS, fibrosis, and chronic rejection. An early study demonstrated that HO-1 expression was elevated in human lung allografts with acute cellular rejection and obliterative bronchiolitis (Lu et al., 2002). Recipient iron overload and hyperferritinemia were also associated with poor prognosis after lung transplantation (Pugh et al., 2005). Administrations for optimizing iron homeostasis before and after lung transplantation were considered. Different iron chelators, namely, deferoxamine, were used in lung preservation and alleviated oxidative stress after transplantation (Lui et al., 2013). Pirfenidone significantly reduced the deposition of iron, increased the expression of HO-1, and alleviated fibrosis and collagen deposits (Liu et al., 2005), demonstrating anti-ferroptosis, anti-fibrotic and antioxidative properties in preventing chronic airway rejection in a rat model.

Targeted inhibition of ferroptosis may be a potential way to protect against ischemia-reperfusion injury. By reducing the oxidative stress response, the cell damage caused by ferroptosis can be avoided to reduce the occurrence of complications.

### Pulmonary Hypertension

Pulmonary hypertension (PH) is usually secondary to a decrease in vessel diameter or an increase in blood flow in the pulmonary vascular bed (Vonk Noordegraaf et al., 2016). Less commonly, PH caused by unknown causes is called primary or idiopathic PH. At present, the dysfunction of pulmonary endothelial and/or vascular smooth muscle cells is considered to be the potential basis for most forms of PH. The proliferation of endothelial and smooth muscle cells leads to thickening of the intima and media with narrowing of the lumina in the entire pulmonary arterial tree. Dysregulated iron homeostasis in the pathological mechanism of PH is not clear and fully recognized. Investigations have established that pulmonary vascular function could be affected by intracellular iron deficiency in pulmonary artery smooth muscle cells (Rhodes et al., 2011), and rats fed on an iron-deficient diet showed substantial pulmonary vascular remodeling and muscularization, medial hypertrophy, perivascular inflammatory cell infiltration strongly associated with rising pulmonary artery pressure (PAH), and right ventricular hypertrophy (Cotroneo et al., 2015), which suggests that iron metabolism participates in the homeostasis of the pulmonary vasculature and that abnormal iron metabolism takes part in the occurrence and development of PH. In contrast, iron deposition was noted in lung tissue sections and was highly correlated with advanced PH in IPF patients (Kim et al., 2010). Moreover, the administration of the iron chelator deferoxamine prevented pulmonary vascular remodeling in chronic hypoxia-induced PH rats (Wong et al., 2012). Whether iron deficiency or overload leads to PH or is merely a consequence is still under debate. Recently, bioinformatic analyses of unregulated ferroptosis-associated genes were performed, and the targets and potential drugs were predicted. However, there were contradictions between the dysregulation and function of some key genes, such as SLC7A11, in these studies (Zhang et al., 2021; Zou et al., 2021). In another recent study, ferroptosis of pulmonary artery endothelial cells (PAECs) was reported to play a critical role in the progression of PH in a monocrotaline-induced model, proven by increased lipid peroxidation, cellular iron concentrations, mitochondrial damage, abnormal expression of GXP4, ferritin heavy chain 1 (FTH1), and NADPH oxidase 4 (NOX4), followed by activation of inflammatory factors and pulmonary artery remodeling. Ferroptosis suppression by ferrostatin-1 postponed lung vascular remodeling and protected right ventricle function in PH (Xie et al., 2022). Based on these findings, a thorough understanding of ferroptosis in PH is needed, and treating PH with medicines based on ferroptosis regulation might be promising in the future.

### Pulmonary Infections

Deaths from pulmonary infections in the form of pneumonia are not rare worldwide. The epithelial surfaces of the lung are consistently exposed to open air containing microbial contaminants, and other common lung diseases and unhealthy lifestyles, such as smoke and alcohol use, render the lung parenchyma vulnerable to virulent organisms.

Although previously discussed, *P. aeruginosa* is associated with infections in CF patients, and it is most commonly seen in the hospital environment. Based on reports, *P. aeruginosa* can express lipoxygenase (pLoxA) after infection, oxidize host arachidonoyl-phosphatidylethanolamine (ETE-PE) to pro-ferroptotic 15-hydroperoxy-arachidonyl-PE (15-HpETE-PE), and cause ferroptosis of human bronchial epithelial cells (Dar et al., 2018). In addition, *P. aeruginosa* can degrade the host GPX4 defense by stimulating lysosomal chaperone-mediated autophagy (CMA) (Dar et al., 2021). Baicalein inhibits LOX-mediated ferroptotic pathways, which may be a possible target for the treatment of respiratory infections (Dar et al., 2022). In another study, knockdown of anoctamin 1 (ANO1 or TMEM16A), antioxidants, such as coenzyme Q10 (CoQ10) and idebenone, and ferrostatin-1 attenuated *Pseudomonas* aeruginosa-induced cell death in CF bronchial epithelial cells (Ousingsawat et al., 2021).

Pulmonary *tuberculosis* (TB) is the major infectious disease within the spectrum of chronic pneumonia that seriously endangers human health. The World Health Organization (WHO) estimates that TB causes 6% of the world’s deaths and is now deteriorating the condition more worldwide. Immunity to *tuberculosis* infection is mainly driven by Th1 cells, which trigger macrophages to kill bacteria (Jasenosky et al., 2015). Re-exposure to *Mycobacterium tuberculosis* (Mtb) or reactivation of the infection in a previously sensitized host stimulates a swift defensive reaction, but hypersensitivity also increases tissue necrosis and tissue destruction. Mtb-induced macrophage death was related to a decrease in GSH and GPX4 levels and an increase in free iron, mitochondrial superoxide, and lipid peroxidation, which indicates that ferroptosis was involved in tissue necrosis in Mtb infection (Amaral et al., 2019). Mtb also increased the expression of HO-1, which may in turn facilitate Mtb survival and growth as a consequence of increased iron availability (Yang et al., 2022). The destruction of macrophages by Mtb and bacterial load were reduced after ferrostatin-1 treatment (Amaral et al., 2019). The primary anti-tuberculosis drugs isoniazid (INH) and rifampicin (LFP) usually exhaust GSH and cause lipid peroxidation and ferroptosis of hepatocytes during liver metabolism. GSH replenishment prevented injury, while iron supplementation augmented the ferroptosis process (Pan et al., 2020). Vitamin E (vit E) was used to intervene in *tuberculosis* patients in clinical trials. The results showed that MDA levels were reduced, and the plasma total antioxidants were improved in *tuberculosis* patients after 2 months of administration (Seyedrezazadeh et al., 2008). This can be explained by the fact that vit E can reduce nonheme iron from ferric iron to ferrous iron to inhibit 15-LOX1 (Hinman et al., 2018), therefore, preventing the ferroptosis pathway regulated by 15-LOX1 (Kagan et al., 2017).

Ferroptosis inhibitors were also investigated for the treatment of resistant pathogens, such as liproxstatin-1, which was proven to interact with lipopolysaccharide in the outer leaflet to disrupt the integrity of the outer membrane of Gram-negative bacteria and potentially serve as an outer membrane permeabilizing compound (Klobucar et al., 2021).

The coronavirus disease 2019 (COVID-19) outbreak caused by severe acute respiratory syndrome coronavirus 2 (SARS-CoV-2) is an ongoing global health emergency, and the pathologic process of the virus has not been completely elucidated. The clinical symptoms are mostly cough, fever, chest distress, muscle aches, fatigue, dyspnea, and headache (Huang et al., 2020a; Wang et al., 2020). Conditions including acute respiratory distress syndrome (ARDS), septic shock, severe metabolic acidosis, and a hypercoagulable state are life-threatening in severe cases with COVID-19, and respiratory failure is the most common cause of death. The pathological lesions include hemoglobinopathy, hypoxia, and hyperferritinemia (Zhao et al., 2020b; Cavezzi et al., 2020; and Colafrancesco et al., 2020). Cellular iron overload is believed to play a pivotal role in COVID-19 infection. Elevated serum ferritin can further aggravate systematic inflammation, which is closely related to poor prognosis (Huang et al., 2020b; Edeas et al., 2020; and Zhou et al., 2020). Considering that the expression of transferrin and the risk of severe cases in male patients were higher than those in female patients and increased with age, this relationship could explain the higher infection rate and mortality in elderly male COVID-19 patients. Transferrin could deliver iron to cells by binding the transferrin receptor and mediating endocytosis. The higher levels of the soluble transferrin receptor in COVID-19 patients confirmed this possibility (Duca et al., 2021). It remains unclear whether hyperferritinemia is a systemic marker or a key modulator in the pathogenesis of COVID-19. Iron is engaged in several biological processes involving DNA, RNA, and ATP synthesis. There is evidence that the replication of SARS-CoV-2 depends on iron-containing enzymes. Regulating host iron metabolism can play an antiviral role and prolong cell survival. Iron is also responsible for SARS-CoV-2 replication, which include key steps, such as ATP hydrolysis (Jia et al., 2019). Moreover, SOD and lipid peroxidation levels were significantly elevated in COVID-19 patients, while GSH and GPX4 levels were decreased (Silvagno et al., 2020). Changes in iron metabolism, GSH depletion, GPX4 inactivation, and upregulation of lipid peroxidation may lead to the hypothesis that ferroptosis may be triggered after SARS-CoV-2 infection, which results in damage to multiple organs. Early treatments with the antioxidants vitamin C (Vit C), vitamin E (Vit E), N-acetylcysteine (NAC) and melatonin (MT) with pentoxifylline were reported to scavenge ROS, supply GSH, and delay the aggression and death of COVID-19 (Jaiswal et al., 2020; Chavarría et al., 2021). Iron chelators, such as deferiprone, deferasirox, deferoxamine exhibited iron chelating, antiviral, and immunomodulatory effects *in vitro* and *in vivo*. Critically ill patients can benefit from orally given deferasirox or intravenously given deferoxamine. Several mechanisms are involved in this process: a. inhibiting viral replication; b. reducing iron availability; c. upregulating B cells; d. increasing neutralizing antibody titer; e. inhibiting endothelial cell inflammation; and f. preventing lung fibrosis and pulmonary recession by reducing pulmonary iron accumulation (Cavezzi et al., 2020; Dalamaga et al., 2020). However, there have been debates on the use of deferoxamine or hepcidin antagonists, whereas it was declared that deferoxamine could also downregulate the expression of hepcidin I (Abobaker, 2021; Garrick and Ghio, 2021). Lactoferrin is a naturally occurring, nontoxic glycoprotein that contains more pertinence to binding iron than transferrin and has demonstrated antiviral efficacy against different kinds of viruses *in vitro*, including SARS-CoV, and might potentially serve as a preventative and adjunct treatment for COVID-19 (Carota et al., 2021). Theoretically, drugs, for instance, methemoglobin reductase, ascorbic acid (Vit C), and GSH were inferred to reduce ferric iron to ferrous iron in hemoglobin to restore its ability to combine with oxygen to alleviate the symptoms of hypoxia in COVID-19 patients (Muhoberac, 2020). In conclusion, reducing cellular iron and replenishing the level of reductants are the most basic treatment methods to lessen tissue injury in COVID-19 patients. These drugs also ought to be investigated in future clinical studies to confirm their safety and effectiveness.

Mucormycosis is caused by a fungus called Zygomycetes. It is a life-threatening opportunistic mycosis that is generally confined to immunocompromised patients, especially those with hematolymphoid malignancies or severe neutropenia, those receiving corticosteroids, or other immunosuppressive drugs, allogeneic stem cell transplant recipients, poorly controlled diabetes mellitus (DM), and those with an iron overload state. Evidence suggests that iron metabolism and fungal endothelial cell interactions play an important role in the pathogenesis of mucormycosis (Hamilos et al., 2011). In a recent report, both infected and recovered COVID-19 patients were promptly infected with mucormycetes (Pasrija and Naime, 2022). The reason might be that COVID-19 patients with elevated serum levels of available iron were susceptible to mucormycosis, and these infections are highly angioinvasive, since the pathogens could acquire iron from the host and interact with endothelial cells lining blood vessels (Pasrija and Naime, 2022). In addition, SARS-CoV-2 increased the susceptibility of patients to mucormycosis by augmenting the virulence factors of the Mucor species. Iron chelator therapy may be advantageous to treat the infection by correcting and inappropriately supplying the fungus with iron. However, there was evidence that the new oral iron chelators deferiprone and deferasirox, better than deferoxamine, could deteriorate the growth of fungi both *in vitro* and in animal models. The iron liberated from deferoxamine was likely transported into the fungus by the high-affinity iron permease, thus, promoting infections. Deferiprone showed the highest antimicrobial activity and tissue penetration capacity, particularly access to the brain (Kontoghiorghes et al., 2010; Álvarez et al., 2013). Melatonin (MT) is an iron chelator, calmodulin blocker, and inhibitor of myeloperoxidase along with an inhibitor of ferroptosis and pyroptosis. By correcting MT deficiency, the enhancement of fungal virulence in COVID-19 patients was alleviated because MT could hinder the iron acquisition of Mucor species and prevent their morphological transformation from yeast to the virulent hyphal form (Sen, 2021).

Aspergillus fumigatus is the most common airborne fungal pathogen and is accountable for invasive aspergillosis in immunocompromised hosts. The acquisition of iron is important for the growth of Aspergillus fumigatus. This fungus synthesizes and secretes triacetylfusarinine C to capture iron and accumulates ferricrocin and hydroxyferricrocin to store iron for hyphae and conidia. Meanwhile, it decreased the expression of the iron importer DMT1 and the transferrin receptor and iron exporter FPN1 (Seifert et al., 2008). In lung transplant recipients, Aspergillus fumigatus infection can be life-threatening. The microhemorrhage-related iron content in the graft might be the main determining factor of invasion and virulence of infection, and progressive graft rejection was related to the increase in ferric iron concentration. Iron chelation, including deferiprone, deferasirox, and deferoxamine, maybe a potential therapy for Aspergillus fumigatus (Hsu et al., 2018), but the effects can be paradoxical, thus, chelators must be chosen carefully.

### Acute Lung Injury/Acute Respiratory Distress Syndrome

The pulmonary infiltrates in ALI are caused by damage to the alveolar-capillary membrane, consisting of the microvascular endothelium and the alveolar epithelium. The acute results of damage include increased vascular permeability and alveolar flooding, impaired diffusion capacity, and extensive surfactant abnormalities. ALI can progress to more severe diffuse alveolar damage and is known as ARDS in the setting of sepsis, severe trauma, or a diffuse lung infection. The clinical features are the emergence of life-threatening respiratory dysfunction, cyanosis, and hypoxemia that is refractory to oxygen therapy and rapidly progresses to multisystem organ failure. Neutrophils and their products, e.g., oxidants, proteases, platelet-activating factor, and leukotrienes, play a crucial role in the pathologic process of ARDS by causing damage to the alveolar-capillary membrane. On the other hand, the endogenous antiproteases, antioxidants, and anti-inflammatory cytokines that counteract the destruction and balance determine the degree of tissue injury and severity of clinical symptoms in ARDS. The characteristic finding in ARDS is the presence of hyaline membranes consisting of fibrin-rich edema fluid admixed with remnants of necrotic epithelial cells, particularly lining the distended alveolar ducts.

In ALI mouse models induced by intestinal ischemia-reperfusion and oleic acid, mitochondrial shrinkage, and mitochondrial membrane rupture were noted in type II alveolar epithelial cells (AEC2). The characteristic indicators of ferroptosis, namely, iron overload, GSH depletion and MDA accumulation, and downregulated GPX4 and ferritin in lung tissue were also detected (Zhou et al., 2019; Dong et al., 2020). In a mouse model of ALI-induced by intestinal ischemia-reperfusion, Nrf2 expression can inhibit ferroptosis *via* modulation of telomerase reverse transcriptase (TERT), HO-1, and SLC7A11 levels (Dong et al., 2020; Dong et al., 2021). In another study, the overexpression of inhibitor of apoptosis stimulating protein of p53 (iASPP) and Nrf2 exhibited therapeutic effects. iASPP inhibited ferroptosis and alleviated tissue injury, depending on Nrf2/hypoxia-inducible factor 1 (HIF1) signal transduction (Li et al., 2020a). Nrf2 upregulation also alleviated GPX4 decreases and attenuated signal transducer and activator of transcription-3 (STAT3) phosphorylation (pSTAT3). STAT3 enhanced the antioxidant capacity through SLC7A11 activation, thereby attenuating the development of ferroptosis during the disease (Qiang et al., 2020). In lipopolysaccharide (LPS)-induced ALI animal models and bronchial epithelial cell lines, the levels of MDA, 4-hydroxynonenal (4-HNE), and total iron were dramatically increased, and the expression levels of SLC7A11 and GPX4 were decreased, indicating that ferroptosis was involved in LPS-induced ALI (Liu et al., 2020). Ferroptosis inhibitors, including ferrostatin-1 and liproxstatin-1, showed a protective effect in these ALI/ARDS models. In addition, Panaxadiol (PX) derived from Panax ginseng root was utilized in LPS-induced ALI/ARDS, and the results showed that PX lessened the pathological lesions in mouse lungs, inhibiting ferroptosis by upregulating the Kelch-like ECH-associated protein 1 (KEAP1)/Nrf2/HO-1 pathway (Li et al., 2021b). Similarly, 4-octyl itaconate (4-OI) and obacunone (OB) were reported to significantly alleviate lung injury, increase GSH and GPX4, and reduce malonaldehyde and lipid peroxidation by activating Nrf2 and HO-1 *in vivo* (He et al., 2022b; Li et al., 2022). Puerarin (PUE) (Xu et al., 2021a), silencing or knockdown of mixed lineage kinase 3 (MLK3) (Chen et al., 2022), lipocalin-2 (Wang et al., 2022b) and Jumonji domain-containing 3 (JMJD3) (Peng et al., 2021), and electroacupuncture (EA) (Zhang et al., 2022) presented novel targets for the treatment of LPS-induced ALI/ARDS. These studies preliminarily confirmed that ferroptosis is intricately connected to ALI/ARDS and that it can be a novel therapeutic target.

### Pulmonary Alveolar Proteinosis

Pulmonary alveolar proteinosis (PAP) is an infrequent disease characterized by the aggregation of surfactant and phospholipids in the distal airways and alveoli. The causative factors in primary PAP are still unknown, but it is postulated to be an autoimmune disorder since the emergence of antibodies to granulocyte-macrophage colony-stimulating factor (GM-CSF). Disturbance of iron homeostasis in epithelial cells and macrophages in the lung has been reported in idiopathic PAP patients. Several early reports demonstrated that the contents of iron, transferrin, transferrin receptor, lactoferrin, and ferritin were remarkably elevated in lavage from PAP patients in comparison with healthy controls, while the concentrations of ascorbate, glutathione, and urate were remarkably lower. The cells of PAP patients accumulated significant iron and ferritin. Immunohistochemistry for the lung tissue revealed the accumulation of ferritin in the lower respiratory tract of PAP patients (Ghio et al., 2008b; Shimizu et al., 2011). This led to the hypothesis that ferroptosis might participate in the development of PAPs; however, there is neither direct evidence to prove the correlation between ferroptosis and PAPs nor intervention experiments to evaluate the effect of medicines based on ferroptosis regulation on PAPs.

### Lung Cancer

Lung cancer has the highest incidence rate and mortality rate in the world. Accumulated studies have found that ferroptosis has a close relationship with lung cancer and tumor metastasis, and lung cancer cells are in a state of ferroptosis inhibition.

a System Xc- (xCT) is a cystine/glutamate antiporter consisting of SLC3A2 and SLC7A11, which is accountable for exporting glutamate and importing cysteine. By upregulating SLC3A2 and SLC7A11, lung cancer cells could enhance their antioxidant effect, inhibit the occurrence of ferroptosis, and increase drug resistance to inducers of ferroptosis, such as imidazole ketone erastin (IKE) and cisplatin (DDP) (Huang et al., 2005; Ma et al., 2021; Tabnak et al., 2021; and Wang et al., 2021).
b The antioxidant enzymes GPXs, GPX8, and GPX4 in particular, have been identified to play an extensive and broad role in the pathological process of cancers (Zhang et al., 2020a). Overexpression of GPX4 promoted the proliferative capacity of lung cancer cells and inhibited ferroptosis, whereas RSL3 hindered GPX4 activity and limited the proliferation, migration, invasion, and angiogenesis of lung cancer cells (Zhang et al., 2020a; Tabnak et al., 2021). Besides, these antioxidants can protect metastasizing cancer cells in both circulations and the metastatic niche to resist ferroptosis (Liu et al., 2021a). Targeting the GPX4 pathway may provide a new strategy for treating lung cancer growth and metastasis.
c The tumor-suppressive activity of p53 has been proposed and proven after decades of intensive study. Mutation of p53 cannot repress SLC7A11 and promote ferroptosis (Jiang et al., 2015b). In addition, the p53 P47S polymorphism, commonly found in people of African descent, is also defective in promoting ferroptosis and repressing tumor development (Leu et al., 2019). Targeting p53 can potentially improve the efficiency of lung cancer treatment by mediating ferroptotic responses.
d Ferroptosis suppressor protein 1 (FSP1) is a ferroptosis inhibitor that is independent of the GPX4 pathway and could suppress ferroptosis *via* CoQ10 (Doll et al., 2019). FSP1 is decorated with cardamom acylation by regulating NADPH to reduce CoQ10, producing lipophilic free radicals to capture free antioxidants to prevent lipid peroxidation to suppress ferroptosis (Doll et al., 2019). High expression of FSP1 could lead to increased resistance in lung cancer cells (Doll et al., 2019).
e MicroRNAs (miRNAs) are a class of noncoding RNAs (ncRNAs) with a length of 18–25 nucleotides that could impede protein translation by regulating their target mRNAs. Tumor suppressor miRNAs are negatively regulated in cancers and usually target oncogenic proteins. Downregulated tumor suppressor miRNAs that induce ferroptosis, such as miR-302a-3p and miR-324-3p, can promote survival and proliferation, and their overexpression sensitizes resistant cells to ferroptosis and increases the sensitivity of chemotherapeutic drugs (Deng et al., 2021; Wei et al., 2021).
f LncRNAs engage in the occurrence and development of non-small-cell lung cancers (NSCLCs) by mediating ferroptosis. LINC00472 (P53RRA) activates the p53 pathway by interacting with Ras GTPase-activating protein-binding protein 1 (G3BP1) and induces ferroptosis (Mao et al., 2018). P53RRA promotes erastin-induced growth inhibition and increases the cellular iron and lipid ROS concentrations in NSCLC cells (Mao et al., 2018). Low expression of P53RRA removed p53 and weakened ferroptosis in NSCLC (Mao et al., 2018). In addition, overexpressed LINC00336 acted as a crucial ferroptosis inhibitor in lung cancer by lowering cellular iron, lipid peroxidation, and mitochondrial superoxide through ELAV-like RNA-binding protein 1 (ELAVL1) interactivity, a novel regulator of ferroptosis (Wang et al., 2019). LINC00336 also served as an endogenous sponge of MIR6852 as a circulating extracellular DNA (ceRNA) to increase cystathionine-β synthase (CBS) expression and inhibit ferroptosis in lung cancer (Wang et al., 2019). Overexpression of LINC00336 limited RSL3-induced ferroptosis in lung adenocarcinoma cells.

These studies established that resistance to ferroptosis was enhanced from multiple aspects in lung cancer and metastasis. The first thing that comes to mind is that the promising ferroptosis inducers erastin and RSL3 can inhibit the biological process of System Xc- and GPXs (Huang et al., 2018; Pan et al., 2019; Zhang et al., 2020b; Liu et al., 2021b). Other signaling pathways can be involved in these ferroptosis inducers. For example, erastin can activate the p53 signaling pathway, subsequently inhibiting the expression of SCL7A11 posttranscriptionally and subsequently inducing ferroptosis (Huang et al., 2018). DDP, a classical chemotherapeutic drug, was recently proven to trigger ferroptosis in NSCLC by inhibiting the activity of the GSH-GPX system and promoting the therapeutic effect together with erastin (Guo et al., 2018). Similarly, an analog of erastin, PRLX93936, can induce ferroptosis *via* GPX4 inhibition when combined with DDP (Liang et al., 2021). Novel ferroptosis inhibitors, such as IKE and sorafenib, can inhibit the cystine/glutamate transporter, exhaust GSH, and increase sensitivity to radiotherapy (Li et al., 2020b; Ye et al., 2020). The local anesthetic levobupivacaine could increase p53 expression, enhance ferroptosis, and inhibit tumor growth in NSCLC (Meng et al., 2021). The dual PI3K/HDAC inhibitor BEBT-908 could activate immunogenic ferroptosis by hyperacetylating and activating p53 in lung cancer cells (Fan et al., 2021). An FSP1 inhibitor (iFSP1) could reverse FSP1-mediated drug resistance by increasing cellular sensitivity to ferroptosis and thus, promoting PCD in lung cancer cells (Doll et al., 2019). The application of erianin (Chen et al., 2020b), sanguinarine (SAG) (Xu et al., 2022), and dihydroisotanshinone I (DT) (Wu et al., 2021) also repressed tumor growth and prevented metastasis *in vivo* and *in vitro*. In addition, targeted delivery of ncRNAs is also considered a promising anticancer strategy (Tabnak et al., 2021), and the application of delicate nanotechnology has recently attracted extensive attention due to its specific physicochemical properties (Chen et al., 2021b). Follow-up research can carry out more effective and precise interventions in the above and other regulatory pathways, regulating cellular sensitivity to ferroptosis, inhibiting the growth and metastasis of lung cancer cells, and prolonging the survival of patients.

## Conclusion and Perspectives

As a unique form of PCD, ferroptosis has received growing attention and interest since its first report in 2012 (Dixon et al., 2012). With the progress of ferroptosis-related research, it has been revealed that ferroptosis plays a crucial role in many pathological changes in lung diseases. Among these findings, the dysregulation of ferroptosis in lung cancer has been more widely and deeply explored (Xu et al., 2021b; Yu et al., 2021). In this review, we provide a current understanding and views of ferroptosis in lung diseases, especially beyond lung cancer, from the aspects of the molecular basis and the corresponding therapeutic significance. Impressive efforts have been made to reveal the potential pathological mechanisms of ferroptosis in diseases, such as COPD, asthma, and pulmonary fibrosis, which provide fresh thinking for the treatment of these diseases in a large number of suffering patients. However, the following questions remain to be addressed for further clinical development of ferroptosis-targeted therapies. First, are there other potential ferroptosis regulators and mechanisms? Current thinking suggests that p53 and GPX4 are two main ferroptosis-regulatory mechanisms but are not mutually exclusive. Second, what is the optimal timing, dose, and route of administration for treatment targeting ferroptosis in specific pathological lung conditions and disease stages? Persistent usage, overdose, and systemic administration of drugs that inactivate ferroptosis might theoretically increase cancer occurrence. Third, what physiological role does ferroptosis play in lung disease? Ferroptosis is involved in the occurrence of asthma and the development of inflammation, thus, some studies have shown that inhibition of ferroptosis can be developed as a therapeutic target, while ferroptosis of eosinophils could result in sensitivity to dexamethasone and relieve the symptoms of asthmatic patients. Hence, an appropriate treatment for different types of lung disease must be carefully chosen. Fourth, in treating lung cancer and metastasis, can we activate ferroptosis specifically in cancer cells without affecting healthy cells? Inhibitions of specific ferroptotic targets are better choices than targeting p53 or GPX4 to preserve their comprehensive influence on tumor suppression and antioxidation in normal cells. It is of great significance and value to further study the pathogenesis of ferroptosis in lung diseases because of insufficient understanding, to determine sensitive biological indicators, and reliable therapeutic targets. More efficient and specific regulation of cellular ferroptosis is pivotal to further investigations.

## References

[B1] Abobaker A. (2021). Reply: Iron Chelation May Harm Patients with COVID-19. Eur. J. Clin. Pharmacol. 77 (2), 267–268. 10.1007/s00228-020-02988-9 32870381 PMC7459945

[B2] Álvarez F. Fernández-Ruiz M. Aguado J. M. (2013). Iron and Invasive Fungal Infection. Rev. Iberoam. Micol. 30 (4), 217–225. 23684655 10.1016/j.riam.2013.04.002

[B3] Amaral E. P. Costa D. L. Namasivayam S. Riteau N. Kamenyeva O. Mittereder L. (2019). A Major Role for Ferroptosis in Mycobacterium Tuberculosis-Induced Cell Death and Tissue Necrosis. J. Exp. Med. 216 (3), 556–570. 10.1084/jem.20181776 30787033 PMC6400546

[B4] Barnes P. J. Burney P. G. Silverman E. K. Celli B. R. Vestbo J. Wedzicha J. A. (2015). Chronic Obstructive Pulmonary Disease. Nat. Rev. Dis. Prim. 1 (1), 15076. 10.1038/nrdp.2015.76 27189863

[B5] Barnes P. J. Baker J. Donnelly L. E. (2019). Cellular Senescence as a Mechanism and Target in Chronic Lung Diseases. Am. J. Respir. Crit. Care Med. 200 (5), 556–564. 10.1164/rccm.201810-1975tr 30860857

[B6] Baz M. A. Ghio A. J. Roggli V. L. Tapson V. F. Piantadosi C. A. (1997). Iron Accumulation in Lung Allografts after Transplantation*. Chest 112 (2), 435–439. 10.1378/chest.112.2.435 9266881

[B7] Carota G. Ronsisvalle S. Panarello F. Tibullo D. Nicolosi A. Li Volti G. (2021). Role of Iron Chelation and Protease Inhibition of Natural Products on COVID-19 Infection. J. Clin. Med. 10 (11). 10.3390/jcm10112306 PMC8198259 34070628

[B8] Cavezzi A. Troiani E. Corrao S. (2020). COVID-19: Hemoglobin, Iron, and Hypoxia beyond Inflammation. A Narrative Review. Clin. Pract. 10 (2), 1271. 10.4081/cp.2020.1271 32509258 PMC7267810

[B9] Chavarría A. P. Valdez Vázquez R. R. Domínguez Cherit J. G. Herrera Bello H. Castillejos Suastegui H. Moreno-Castañeda L. (2021). Antioxidants and Pentoxifylline as Coadjuvant Measures to Standard Therapy to Improve Prognosis of Patients with Pneumonia by COVID-19. Comput. Struct. Biotechnol. J. 19, 1379–1390. 10.1016/j.csbj.2021.02.009 33680348 PMC7910139

[B10] Chen K. Zhang S. Jiao J. Zhao S. (2021). Ferroptosis and its Potential Role in Lung Cancer: Updated Evidence from Pathogenesis to Therapy. Jir Vol. 14, 7079–7090. 10.2147/jir.s347955 PMC8709579 34992407

[B11] Chen L.-D. Huang Z.-W. Huang Y.-Z. Huang J.-F. Zhang Z.-P. Lin X.-J. (2021). Untargeted Metabolomic Profiling of Liver in a Chronic Intermittent Hypoxia Mouse Model. Front. Physiol. 12, 701035. 10.3389/fphys.2021.701035 34305653 PMC8298499

[B12] Chen L.-D. Wu R.-H. Huang Y.-Z. Chen M.-X. Zeng A.-M. Zhuo G.-f. (2020). The Role of Ferroptosis in Chronic Intermittent Hypoxia-Induced Liver Injury in Rats. Sleep. Breath. 24 (4), 1767–1773. 10.1007/s11325-020-02091-4 32361960

[B13] Chen P. Wu Q. Feng J. Yan L. Sun Y. Liu S. (2020). Erianin, a Novel Dibenzyl Compound in Dendrobium Extract, Inhibits Lung Cancer Cell Growth and Migration via Calcium/calmodulin-dependent Ferroptosis. Sig Transduct. Target Ther. 5 (1), 51. 10.1038/s41392-020-0149-3 PMC7205607 32382060

[B14] Chen X. Qi G. Fang F. Miao Y. Wang L. (2022). Silence of MLK3 Alleviates Lipopolysaccharide-Induced Lung Epithelial Cell Injury via Inhibiting P53-Mediated Ferroptosis. J. Mol. Histol. 53 (2), 503–510. 10.1007/s10735-022-10064-y 35247112

[B15] Cheng H. Feng D. Li X. Gao L. Tang S. Liu W. (2021). Iron Deposition-Induced Ferroptosis in Alveolar Type II Cells Promotes the Development of Pulmonary Fibrosis. Biochimica Biophysica Acta (BBA) - Mol. Basis Dis. 1867 (12), 166204. 10.1016/j.bbadis.2021.166204 34175430

[B16] Colafrancesco S. Alessandri C. Conti F. Priori R. (2020). COVID-19 Gone Bad: A New Character in the Spectrum of the Hyperferritinemic Syndrome? Autoimmun. Rev. 19 (7), 102573. 10.1016/j.autrev.2020.102573 32387470 PMC7199723

[B17] Cotroneo E. Ashek A. Wang L. Wharton J. Dubois O. Bozorgi S. (2015). Iron Homeostasis and Pulmonary Hypertension. Circ. Res. 116 (10), 1680–1690. 10.1161/circresaha.116.305265 25767292

[B18] Dalamaga M. Karampela I. Mantzoros C. S. (2020). Commentary: Could Iron Chelators Prove to Be Useful as an Adjunct to COVID-19 Treatment Regimens? Metabolism 108, 154260. 10.1016/j.metabol.2020.154260 32418885 PMC7207125

[B19] Dar H. H. Epperly M. W. Tyurin V. A. Amoscato A. A. Anthonymuthu T. S. Souryavong A. B. (2022). *P. aeruginosa* Augments Irradiation Injury via 15-Lipoxygenase-Catalyzed Generation of 15-HpETE-PE and Induction of Theft-Ferroptosis. JCI Insight 7 (4). 10.1172/jci.insight.156013 PMC8876480 35041620

[B20] Dar H. H. Anthonymuthu T. S. Ponomareva L. A. Souryavong A. B. Shurin G. V. Kapralov A. O. (2021). A New Thiol-independent Mechanism of Epithelial Host Defense against *Pseudomonas aeruginosa*: iNOS/NO Sabotage of Theft-Ferroptosis. Redox Biol. 45, 102045. 10.1016/j.redox.2021.102045 34167028 PMC8227829

[B21] Dar H. H. Tyurina Y. Y. Mikulska-Ruminska K. Shrivastava I. Ting H.-C. Tyurin V. A. (2018). *Pseudomonas aeruginosa* Utilizes Host Polyunsaturated Phosphatidylethanolamines to Trigger Theft-Ferroptosis in Bronchial Epithelium. J. Clin. Invest. 128 (10), 4639–4653. 10.1172/jci99490 30198910 PMC6159971

[B22] Deng S.-h. Wu D.-m. Li L. Liu T. Zhang T. Li J. (2021). miR-324-3p Reverses Cisplatin Resistance by Inducing GPX4-Mediated Ferroptosis in Lung Adenocarcinoma Cell Line A549. Biochem. Biophysical Res. Commun. 549, 54–60. 10.1016/j.bbrc.2021.02.077 33662669

[B23] Dixon S. J. Lemberg K. M. Lamprecht M. R. Skouta R. Zaitsev E. M. Gleason C. E. (2012). Ferroptosis: an Iron-dependent Form of Nonapoptotic Cell Death. Cell. 149 (5), 1060–1072. 10.1016/j.cell.2012.03.042 22632970 PMC3367386

[B24] Dodson M. Castro-Portuguez R. Zhang D. D. (2019). NRF2 Plays a Critical Role in Mitigating Lipid Peroxidation and Ferroptosis. Redox Biol. 23, 101107. 10.1016/j.redox.2019.101107 30692038 PMC6859567

[B25] Doll S. Freitas F. P. Shah R. Aldrovandi M. da Silva M. C. Ingold I. (2019). FSP1 Is a Glutathione-independent Ferroptosis Suppressor. Nature 575 (7784), 693–698. 10.1038/s41586-019-1707-0 31634899

[B26] Doll S. Proneth B. Tyurina Y. Y. Panzilius E. Kobayashi S. Ingold I. (2017). ACSL4 Dictates Ferroptosis Sensitivity by Shaping Cellular Lipid Composition. Nat. Chem. Biol. 13 (1), 91–98. 10.1038/nchembio.2239 27842070 PMC5610546

[B27] Dong H. Qiang Z. Chai D. Peng J. Xia Y. Hu R. (2020). Nrf2 Inhibits Ferroptosis and Protects against Acute Lung Injury Due to Intestinal Ischemia Reperfusion via Regulating SLC7A11 and HO-1. Aging 12 (13), 12943–12959. 10.18632/aging.103378 32601262 PMC7377827

[B28] Dong H. Xia Y. Jin S. Xue C. Wang Y. Hu R. (2021). Nrf2 Attenuates Ferroptosis-Mediated IIR-ALI by Modulating TERT and SLC7A11. Cell. Death Dis. 12 (11), 1027. 10.1038/s41419-021-04307-1 34716298 PMC8556385

[B29] Dowdle W. E. Nyfeler B. Nagel J. Elling R. A. Liu S. Triantafellow E. (2014). Selective VPS34 Inhibitor Blocks Autophagy and Uncovers a Role for NCOA4 in Ferritin Degradation and Iron Homeostasis *In Vivo* . Nat. Cell. Biol. 16 (11), 1069–1079. 10.1038/ncb3053 25327288

[B30] Duca L. Ottolenghi S. Coppola S. Rinaldo R. Dei Cas M. Rubino F. M. (2021). Differential Redox State and Iron Regulation in Chronic Obstructive Pulmonary Disease, Acute Respiratory Distress Syndrome and Coronavirus Disease 2019. Antioxidants (Basel) 10 (9). 10.3390/antiox10091460 PMC8470076 34573092

[B31] Edeas M. Saleh J. Peyssonnaux C. (2020). Iron: Innocent Bystander or Vicious Culprit in COVID-19 Pathogenesis? Int. J. Infect. Dis. 97, 303–305. 10.1016/j.ijid.2020.05.110 32497811 PMC7264936

[B32] Fan F. Liu P. Bao R. Chen J. Zhou M. Mo Z. (2021). A Dual PI3K/HDAC Inhibitor Induces Immunogenic Ferroptosis to Potentiate Cancer Immune Checkpoint Therapy. Cancer Res. 81 (24), 6233–6245. 10.1158/0008-5472.can-21-1547 34711611

[B33] Forcina G. C. Dixon S. J. (2019). GPX4 at the Crossroads of Lipid Homeostasis and Ferroptosis. Proteomics 19 (18), 1800311. 10.1002/pmic.201800311 30888116

[B34] Garrick M. D. Ghio A. J. (2021). Iron Chelation May Harm Patients with COVID-19. Eur. J. Clin. Pharmacol. 77 (2), 265–266. 10.1007/s00228-020-02987-w 32870379 PMC7459091

[B35] Ghio A. J. Hilborn E. D. Stonehuerner J. G. Dailey L. A. Carter J. D. Richards J. H. (2008). Particulate Matter in Cigarette Smoke Alters Iron Homeostasis to Produce a Biological Effect. Am. J. Respir. Crit. Care Med. 178 (11), 1130–1138. 10.1164/rccm.200802-334oc 18723436

[B36] Ghio A. J. Stonehuerner J. G. Richards J. H. Crissman K. M. Roggli V. L. Piantadosi C. A. (2008). Iron Homeostasis and Oxidative Stress in Idiopathic Pulmonary Alveolar Proteinosis: a Case-Control Study. Respir. Res. 9 (1), 10. 10.1186/1465-9921-9-10 18215276 PMC2265287

[B37] Gong Y. Wang N. Liu N. Dong H. (2019). Lipid Peroxidation and GPX4 Inhibition Are Common Causes for Myofibroblast Differentiation and Ferroptosis. DNA Cell. Biol. 38 (7), 725–733. 10.1089/dna.2018.4541 31140862

[B38] Guo J. Xu B. Han Q. Zhou H. Xia Y. Gong C. (2018). Ferroptosis: A Novel Anti-tumor Action for Cisplatin. Cancer Res. Treat. 50 (2), 445–460. 10.4143/crt.2016.572 28494534 PMC5912137

[B39] Hamilos G. Samonis G. Kontoyiannis D. P. (2011). Pulmonary Mucormycosis. Semin. Respir. Crit. Care Med. 32 (6), 693–702. 10.1055/s-0031-1295717 22167397

[B40] Hammad H. Lambrecht B. N. (2021). The Basic Immunology of Asthma. Cell. 184 (6), 1469–1485. 10.1016/j.cell.2021.02.016 33711259

[B41] Han Y. Ye L. Du F. Ye M. Li C. Zhu X. (2021). Iron Metabolism Regulation of Epithelial-Mesenchymal Transition in Idiopathic Pulmonary Fibrosis. Ann. Transl. Med. 9 (24), 1755. 10.21037/atm-21-5404 35071449 PMC8756254

[B42] He J. Li X. Yu M. (2021). Bioinformatics Analysis Identifies Potential Ferroptosis Key Genes in the Pathogenesis of Pulmonary Fibrosis. Front. Genet. 12, 788417. 10.3389/fgene.2021.788417 35069688 PMC8770739

[B43] He R. Liu B. Xiong R. Geng B. Meng H. Lin W. (2022). Itaconate Inhibits Ferroptosis of Macrophage via Nrf2 Pathways against Sepsis-Induced Acute Lung Injury. Cell. Death Discov. 8 (1), 43. 10.1038/s41420-021-00807-3 35110526 PMC8810876

[B44] He Y. Shang Y. Li Y. Wang M. Yu D. Yang Y. (2022). An 8-Ferroptosis-Related Genes Signature from Bronchoalveolar Lavage Fluid for Prognosis in Patients with Idiopathic Pulmonary Fibrosis. BMC Pulm. Med. 22 (1), 15. 10.1186/s12890-021-01799-7 34983465 PMC8728942

[B45] Hinman A. Holst C. R. Latham J. C. Bruegger J. J. Ulas G. McCusker K. P. (2018). Vitamin E Hydroquinone Is an Endogenous Regulator of Ferroptosis via Redox Control of 15-lipoxygenase. PLoS One 13 (8), e0201369. 10.1371/journal.pone.0201369 30110365 PMC6093661

[B46] Hsu J. L. Manouvakhova O. V. Clemons K. V. Inayathullah M. Tu A. B. Sobel R. A. (2018). Microhemorrhage-associated Tissue Iron Enhances the Risk for Aspergillus fumigatus Invasion in a Mouse Model of Airway Transplantation. Sci. Transl. Med. 10 (429). 10.1126/scitranslmed.aag2616 PMC5841257 29467298

[B47] Huang C. Yang M. Deng J. Li P. Su W. Jiang R. (2018). Upregulation and Activation of P53 by Erastin-induced R-eactive O-xygen S-pecies C-ontribute to C-ytotoxic and C-ytostatic E-ffects in A549 L-ung C-ancer C-ells. Oncol. Rep. 40 (4), 2363–2370. 10.3892/or.2018.6585 30066882

[B48] Huang C. Wang Y. Li X. Ren L. Zhao J. Hu Y. (2020). Clinical Features of Patients Infected with 2019 Novel Coronavirus in Wuhan, China. Lancet 395 (10223), 497–506. 10.1016/s0140-6736(20)30183-5 31986264 PMC7159299

[B49] Huang I. Pranata R. Lim M. A. Oehadian A. Alisjahbana B. (2020). C-reactive Protein, Procalcitonin, D-Dimer, and Ferritin in Severe Coronavirus Disease-2019: a Meta-Analysis. Ther. Adv. Respir. Dis. 14, 1753466620937175. 10.1177/1753466620937175 32615866 PMC7336828

[B50] Huang Y. Dai Z. Barbacioru C. Sadée W. (2005). Cystine-glutamate Transporter SLC7A11 in Cancer Chemosensitivity and Chemoresistance. Cancer Res. 65 (16), 7446–7454. 10.1158/0008-5472.can-04-4267 16103098

[B51] Jaiswal N. Bhatnagar M. Shah H. (2020). N-acetycysteine: A Potential Therapeutic Agent in COVID-19 Infection. Med. Hypotheses 144, 110133. 10.1016/j.mehy.2020.110133 32758904 PMC7380211

[B52] Jasenosky L. D. Scriba T. J. Hanekom W. A. Goldfeld A. E. (2015). T Cells and Adaptive Immunity toMycobacterium Tuberculosisin Humans. Immunol. Rev. 264 (1), 74–87. 10.1111/imr.12274 25703553

[B53] Jia Z. Yan L. Ren Z. Wu L. Wang J. Guo J. (2019). Delicate Structural Coordination of the Severe Acute Respiratory Syndrome Coronavirus Nsp13 upon ATP Hydrolysis. Nucleic Acids Res. 47 (12), 6538–6550. 10.1093/nar/gkz409 31131400 PMC6614802

[B54] Jiang L. Hickman J. H. Wang S.-J. Gu W. (2015). Dynamic Roles of P53-Mediated Metabolic Activities in ROS-Induced Stress Responses. Cell. Cycle 14 (18), 2881–2885. 10.1080/15384101.2015.1068479 26218928 PMC4825584

[B55] Jiang L. Kon N. Li T. Wang S.-J. Su T. Hibshoosh H. (2015). Ferroptosis as a P53-Mediated Activity during Tumour Suppression. Nature 520 (7545), 57–62. 10.1038/nature14344 25799988 PMC4455927

[B56] Kagan V. E. Mao G. Qu F. Angeli J. P. F. Doll S. Croix C. S. (2017). Oxidized Arachidonic and Adrenic PEs Navigate Cells to Ferroptosis. Nat. Chem. Biol. 13 (1), 81–90. 10.1038/nchembio.2238 27842066 PMC5506843

[B57] Kim K.-H. Maldonado F. Ryu J. H. Eiken P. W. Hartman T. E. Bartholmai B. J. (2010). Iron Deposition and Increased Alveolar Septal Capillary Density in Nonfibrotic Lung Tissue Are Associated with Pulmonary Hypertension in Idiopathic Pulmonary Fibrosis. Respir. Res. 11 (1), 37. 10.1186/1465-9921-11-37 20398288 PMC2867975

[B58] Klobucar K. Côté J.-P. French S. Borrillo L. Guo A. B. Y. Serrano-Wu M. H. (2021). Chemical Screen for Vancomycin Antagonism Uncovers Probes of the Gram-Negative Outer Membrane. ACS Chem. Biol. 16 (5), 929–942. 10.1021/acschembio.1c00179 33974796

[B59] Kontoghiorghes G. J. Kolnagou A. Skiada A. Petrikkos G. (2010). The Role of Iron and Chelators on Infections in Iron Overload and Non Iron Loaded Conditions: Prospects for the Design of New Antimicrobial Therapies. Hemoglobin 34 (3), 227–239. 10.3109/03630269.2010.483662 20524813

[B60] Lederer D. J. Martinez F. J. Martinez F. J. (2018). Idiopathic Pulmonary Fibrosis. N. Engl. J. Med. 378 (19), 1811–1823. 10.1056/nejmra1705751 29742380

[B61] Leu J. I.-J. Murphy M. E. George D. L. (2019). Mechanistic Basis for Impaired Ferroptosis in Cells Expressing the African-Centric S47 Variant of P53. Proc. Natl. Acad. Sci. U.S.A. 116 (17), 8390–8396. 10.1073/pnas.1821277116 30962386 PMC6486733

[B62] Li J. Deng S.-h. Li J. Li L. Zhang F. Zou Y. (2022). Obacunone Alleviates Ferroptosis during Lipopolysaccharide-Induced Acute Lung Injury by Upregulating Nrf2-dependent Antioxidant Responses. Cell. Mol. Biol. Lett. 27 (1), 29. 10.1186/s11658-022-00318-8 35305560 PMC8933916

[B63] Li J. Lu K. Sun F. Tan S. Zhang X. Sheng W. (2021). Panaxydol Attenuates Ferroptosis against LPS-Induced Acute Lung Injury in Mice by Keap1-Nrf2/HO-1 Pathway. J. Transl. Med. 19 (1), 96. 10.1186/s12967-021-02745-1 33653364 PMC7927246

[B64] Li M. Wang K. Zhang Y. Fan M. Li A. Zhou J. (2021). Ferroptosis-Related Genes in Bronchoalveolar Lavage Fluid Serves as Prognostic Biomarkers for Idiopathic Pulmonary Fibrosis. Front. Med. 8, 693959. 10.3389/fmed.2021.693959 PMC8520927 34671612

[B65] Li X. Duan L. Yuan S. Zhuang X. Qiao T. He J. (2019). Ferroptosis Inhibitor Alleviates Radiation-Induced Lung Fibrosis (RILF) via Down-Regulation of TGF-Β1. J. Inflamm. 16, 11. 10.1186/s12950-019-0216-0 PMC6542066 31160885

[B66] Li Y. Yan H. Xu X. Liu H. Wu C. Zhao L. (2020). Erastin/sorafenib Induces Cisplatin-Resistant Non-small Cell Lung Cancer Cell Ferroptosis through Inhibition of the Nrf2/xCT Pathway. Oncol. Lett. 19 (1), 323–333. 10.3892/ol.2019.11066 31897145 PMC6923844

[B67] Li Y. Cao Y. Xiao J. Shang J. Tan Q. Ping F. (2020). Inhibitor of Apoptosis-Stimulating Protein of P53 Inhibits Ferroptosis and Alleviates Intestinal Ischemia/reperfusion-Induced Acute Lung Injury. Cell. Death Differ. 27 (9), 2635–2650. 10.1038/s41418-020-0528-x 32203170 PMC7429834

[B68] Lian N. Zhang Q. Chen J. Chen M. Huang J. Lin Q. (2021). The Role of Ferroptosis in Bronchoalveolar Epithelial Cell Injury Induced by Cigarette Smoke Extract. Front. Physiol. 12, 751206. 10.3389/fphys.2021.751206 34658933 PMC8511776

[B69] Liang Z. Zhao W. Li X. Wang L. Meng L. Yu R. (2021). Cisplatin Synergizes with PRLX93936 to Induce Ferroptosis in Non-small Cell Lung Cancer Cells. Biochem. Biophysical Res. Commun. 569, 79–85. 10.1016/j.bbrc.2021.06.088 34237431

[B70] Liu H. Drew P. Cheng Y. Visner G. A. (2005). Pirfenidone Inhibits Inflammatory Responses and Ameliorates Allograft Injury in a Rat Lung Transplant Model. J. Thorac. Cardiovasc. Surg. 130 (3), 852–858. 10.1016/j.jtcvs.2005.04.012 16153939

[B71] Liu P. Feng Y. Li H. Chen X. Wang G. Xu S. (2020). Ferrostatin-1 Alleviates Lipopolysaccharide-Induced Acute Lung Injury via Inhibiting Ferroptosis. Cell. Mol. Biol. Lett. 25, 10. 10.1186/s11658-020-00205-0 32161620 PMC7045739

[B72] Liu T. Xu P. Ke S. Dong H. Zhan M. Hu Q. (2022). Histone Methyltransferase SETDB1 Inhibits TGF-β-Induced Epithelial-Mesenchymal Transition in Pulmonary Fibrosis by Regulating SNAI1 Expression and the Ferroptosis Signaling Pathway. Archives Biochem. Biophysics 715, 109087. 10.1016/j.abb.2021.109087 34801472

[B73] Liu W. Zhou Y. Duan W. Song J. Wei S. Xia S. (2021). Glutathione Peroxidase 4-dependent Glutathione High-Consumption Drives Acquired Platinum Chemoresistance in Lung Cancer-Derived Brain Metastasis. Clin. Transl. Med. 11 (9), e517. 10.1002/ctm2.517 34586745 PMC8473645

[B74] Liu W. Chakraborty B. Safi R. Kazmin D. Chang C.-y. McDonnell D. P. (2021). Dysregulated Cholesterol Homeostasis Results in Resistance to Ferroptosis Increasing Tumorigenicity and Metastasis in Cancer. Nat. Commun. 12 (1), 5103. 10.1038/s41467-021-25354-4 34429409 PMC8385107

[B75] Liu X. Ma Y. Luo L. Zong D. Li H. Zeng Z. (2022). Dihydroquercetin Suppresses Cigarette Smoke Induced Ferroptosis in the Pathogenesis of Chronic Obstructive Pulmonary Disease by Activating Nrf2-Mediated Pathway. Phytomedicine 96, 153894. 10.1016/j.phymed.2021.153894 34942457

[B76] Liu Y. Gu W. (2022). p53 in Ferroptosis Regulation: the New Weapon for the Old Guardian. Cell. Death Differ. 29 (5), 895–910. 10.1038/s41418-022-00943-y 35087226 PMC9091200

[B77] Liu Y. Gu W. (2021). The Complexity of P53-Mediated Metabolic Regulation in Tumor Suppression. Semin. Cancer Biol. 10.1016/j.semcancer.2021.03.010 PMC8473587 33785447

[B78] Lu F. Zander D. S. Visner G. A. (2002). Increased Expression of Heme Oxygenase-1 in Human Lung Transplantation. J. Heart Lung Transplant. 21 (10), 1120–1126. 10.1016/s1053-2498(02)00423-0 12398878

[B79] Lui G. Y. L. Obeidy P. Ford S. J. Tselepis C. Sharp D. M. Jansson P. J. (2013). The Iron Chelator, Deferasirox, as a Novel Strategy for Cancer Treatment: Oral Activity against Human Lung Tumor Xenografts and Molecular Mechanism of Action. Mol. Pharmacol. 83 (1), 179–190. 10.1124/mol.112.081893 23074173

[B80] Ma L. Zhang X. Yu K. Xu X. Chen T. Shi Y. (2021). Targeting SLC3A2 Subunit of System XC− Is Essential for m6A Reader YTHDC2 to Be an Endogenous Ferroptosis Inducer in Lung Adenocarcinoma. Free Radic. Biol. Med. 168, 25–43. 10.1016/j.freeradbiomed.2021.03.023 33785413

[B81] Ma X. Yan W. He N. (2022). Lidocaine Attenuates Hypoxia/reoxygenation-Induced Inflammation, Apoptosis and Ferroptosis in Lung Epithelial Cells by Regulating the P38 MAPK Pathway. Mol. Med. Rep. 25 (5). 10.3892/mmr.2022.12666 PMC8941375 35244190

[B82] Maniam P. Essilfie A.-T. Kalimutho M. Ling D. Frazer D. M. Phipps S. (2021). Increased Susceptibility of Cystic Fibrosis Airway Epithelial Cells to Ferroptosis. Biol. Res. 54 (1), 38. 10.1186/s40659-021-00361-3 34903297 PMC8670191

[B83] Mao C. Wang X. Liu Y. Wang M. Yan B. Jiang Y. (2018). A G3BP1-Interacting lncRNA Promotes Ferroptosis and Apoptosis in Cancer via Nuclear Sequestration of P53. Cancer Res. 78 (13), 3484–3496. 10.1158/0008-5472.CAN-17-3454 29588351 PMC8073197

[B84] Meng M. Huang M. Liu C. Wang J. Ren W. Cui S. (2021). Local Anesthetic Levobupivacaine Induces Ferroptosis and Inhibits Progression by Up-Regulating P53 in Non-small Cell Lung Cancer. Aging 13. 10.18632/aging.203138 34175840

[B85] Mizumura K. Gon Y. (2021). Iron-Regulated Reactive Oxygen Species Production and Programmed Cell Death in Chronic Obstructive Pulmonary Disease. Antioxidants (Basel) 10 (10). 10.3390/antiox10101569 PMC8533398 34679704

[B86] Muhoberac B. B. (2020). What Can Cellular Redox, Iron, and Reactive Oxygen Species Suggest about the Mechanisms and Potential Therapy of COVID-19? Front. Cell. Infect. Microbiol. 10, 569709. 10.3389/fcimb.2020.569709 33381464 PMC7767833

[B87] Nagasaki T. Schuyler A. J. Zhao J. Samovich S. N. Yamada K. Deng Y. (2022). 15LO1 Dictates Glutathione Redox Changes in Asthmatic Airway Epithelium to Worsen Type 2 Inflammation. J. Clin. Invest. 132 (1). 10.1172/JCI151685 PMC8718153 34762602

[B88] Ousingsawat J. Schreiber R. Gulbins E. Kamler M. Kunzelmann K. (2021). *P. aeruginosa* Induced Lipid Peroxidation Causes Ferroptotic Cell Death in Airways. Cell. Physiol. Biochem. 55 (5), 590–604. 10.33594/000000437 34637202

[B89] Pan X. Lin Z. Jiang D. Yu Y. Yang D. Zhou H. (2019). Erastin Decreases Radioresistance of NSCLC Cells Partially by Inducing GPX4-Mediated Ferroptosis. Oncol. Lett. 17 (3), 3001–3008. 10.3892/ol.2019.9888 30854078 PMC6365906

[B90] Pan Y. Tang P. Cao J. Song Q. Zhu L. Ma S. (2020). Lipid Peroxidation Aggravates Anti-tuberculosis Drug-Induced Liver Injury: Evidence of Ferroptosis Induction. Biochem. Biophysical Res. Commun. 533 (4), 1512–1518. 10.1016/j.bbrc.2020.09.140 33121683

[B91] Papi A. Brightling C. Pedersen S. E. Reddel H. K. (2018). Asthma. Lancet 391 (10122), 783–800. 10.1016/s0140-6736(17)33311-1 29273246

[B92] Park E.-J. Park Y.-J. Lee S. J. Lee K. Yoon C. (2019). Whole Cigarette Smoke Condensates Induce Ferroptosis in Human Bronchial Epithelial Cells. Toxicol. Lett. 303, 55–66. 10.1016/j.toxlet.2018.12.007 30579903

[B93] Pasrija R. Naime M. (2022). Resolving the Equation between Mucormycosis and COVID-19 Disease. Mol. Biol. Rep. 49 (4), 3349–3356. 10.1007/s11033-021-07085-3 35064406 PMC8782700

[B94] Patel A. R. Patel A. R. Singh S. Singh S. Khawaja I. (2019). The Association between Obstructive Sleep Apnea and Arrhythmias. Cureus 11 (11), e4429–itc96. 10.7759/cureus.4429 31245216 PMC6559391

[B95] Peng J. Fan B. Bao C. Jing C. (2021). JMJD3 Deficiency Alleviates Lipopolysaccharide-induced A-cute L-ung I-njury by I-nhibiting A-lveolar E-pithelial F-erroptosis in a Nrf2-dependent M-anner. Mol. Med. Rep. 24 (5). 10.3892/mmr.2021.12447 34542160

[B96] Prentice B. J. Jaffe A. Hameed S. Verge C. F. Waters S. Widger J. (2021). Cystic Fibrosis-Related Diabetes and Lung Disease: an Update. Eur. Respir. Rev. 30 (159). 10.1183/16000617.0293-2020 PMC9488640 33597125

[B97] Pugh C. Hathwar V. Richards J. H. Stonehuerner J. Ghio A. J. (2005). Disruption of Iron Homeostasis in the Lungs of Transplant Patients. J. Heart Lung Transplant. 24 (11), 1821–1827. 10.1016/j.healun.2005.03.016 16297788

[B98] Qiang Z. Dong H. Xia Y. Chai D. Hu R. Jiang H. (2020). Nrf2 and STAT3 Alleviates Ferroptosis-Mediated IIR-ALI by Regulating SLC7A11. Oxid. Med. Cell. Longev. 2020, 5146982. 10.1155/2020/5146982 33014271 PMC7520693

[B99] Rashidipour N. Karami-Mohajeri S. Mandegary A. Mohammadinejad R. Wong A. Mohit M. (2020). Where Ferroptosis Inhibitors and Paraquat Detoxification Mechanisms Intersect, Exploring Possible Treatment Strategies. Toxicology 433-434, 152407. 10.1016/j.tox.2020.152407 32061663

[B100] Ratjen F. Döring G. (2003). Cystic Fibrosis. Lancet 361 (9358), 681–689. 10.1016/s0140-6736(03)12567-6 12606185

[B101] Rhodes C. J. Wharton J. Howard L. Gibbs J. S. R. Vonk-Noordegraaf A. Wilkins M. R. (2011). Iron Deficiency in Pulmonary Arterial Hypertension: a Potential Therapeutic Target. Eur. Respir. J. 38 (6), 1453–1460. 10.1183/09031936.00037711 21478213

[B102] Seifert M. Nairz M. Schroll A. Schrettl M. Haas H. Weiss G. (2008). Effects of the Aspergillus fumigatus Siderophore Systems on the Regulation of Macrophage Immune Effector Pathways and Iron Homeostasis. Immunobiology 213 (9-10), 767–778. 10.1016/j.imbio.2008.07.010 18926292

[B103] Sen A. (2021). Deficient Synthesis of Melatonin in COVID-19 Can Impair the Resistance of Coronavirus Patients to Mucormycosis. Med. Hypotheses 158, 110722. 10.1016/j.mehy.2021.110722 34753008 PMC8553412

[B104] Seyedrezazadeh E. Ostadrahimi A. Mahboob S. Assadi Y. Ghaemmagami J. Pourmogaddam M. (2008). Effect of Vitamin E and Selenium Supplementation on Oxidative Stress Status in Pulmonary Tuberculosis Patients. Respirology 13 (2), 294–298. 10.1111/j.1440-1843.2007.01200.x 18339032

[B105] Shimizu Y. Matsuzaki S. Dobashi K. Yanagitani N. Satoh T. Koka M. (2011). Elemental Analysis of Lung Tissue Particles and Intracellular Iron Content of Alveolar Macrophages in Pulmonary Alveolar Proteinosis. Respir. Res. 12 (1), 88. 10.1186/1465-9921-12-88 21718529 PMC3141423

[B106] Silvagno F. Vernone A. Pescarmona G. P. (2020). The Role of Glutathione in Protecting against the Severe Inflammatory Response Triggered by COVID-19. Antioxidants (Basel) 9 (7). 10.3390/antiox9070624 PMC7402141 32708578

[B107] Stockwell B. R. Jiang X. Gu W. (2020). Emerging Mechanisms and Disease Relevance of Ferroptosis. Trends Cell. Biol. 30 (6), 478–490. 10.1016/j.tcb.2020.02.009 32413317 PMC7230071

[B108] Sun L. Dong H. Zhang W. Wang N. Ni N. Bai X. (2021). Lipid Peroxidation, GSH Depletion, and SLC7A11 Inhibition Are Common Causes of EMT and Ferroptosis in A549 Cells, but Different in Specific Mechanisms. DNA Cell. Biol. 40 (2), 172–183. 10.1089/dna.2020.5730 33351681

[B109] Sun X. Ou Z. Chen R. Niu X. Chen D. Kang R. (2016). Activation of the P62-Keap1-NRF2 Pathway Protects against Ferroptosis in Hepatocellular Carcinoma Cells. Hepatology 63 (1), 173–184. 10.1002/hep.28251 26403645 PMC4688087

[B110] Tabnak P. HajiEsmailPoor Z. Soraneh S. (2021). Ferroptosis in Lung Cancer: From Molecular Mechanisms to Prognostic and Therapeutic Opportunities. Front. Oncol. 11, 792827. 10.3389/fonc.2021.792827 34926310 PMC8674733

[B111] Takahashi M. Mizumura K. Gon Y. Shimizu T. Kozu Y. Shikano S. (2021). Iron-Dependent Mitochondrial Dysfunction Contributes to the Pathogenesis of Pulmonary Fibrosis. Front. Pharmacol. 12, 643980. 10.3389/fphar.2021.643980 35058772 PMC8765595

[B112] Tang W. Dong M. Teng F. Cui J. Zhu X. Wang W. (2021). Environmental Allergens House Dust Mite-induced A-sthma I-s A-ssociated with F-erroptosis in the L-ungs. Exp. Ther. Med. 22 (6), 1483. 10.3892/etm.2021.10918 34765024 PMC8576623

[B113] Tang W. Dong M. Teng F. Cui J. Zhu X. Wang W. (2021). TMT-based Quantitative Proteomics Reveals Suppression of SLC3A2 and ATP1A3 Expression Contributes to the Inhibitory Role of Acupuncture on Airway Inflammation in an OVA-Induced Mouse Asthma Model. Biomed. Pharmacother. 134, 111001. 10.1016/j.biopha.2020.111001 33341053

[B114] Tang X. Li Z. Yu Z. Li J. Zhang J. Wan N. (2021). Effect of Curcumin on Lung Epithelial Injury and Ferroptosis Induced by Cigarette Smoke. Hum. Exp. Toxicol. 40 (12_Suppl. l), S753–s762. 10.1177/09603271211059497 34787501

[B115] Vonk Noordegraaf A. Groeneveldt J. A. Bogaard H. J. (2016). Pulmonary Hypertension. Eur. Respir. Rev. 25 (139), 4–11. 10.1183/16000617.0096-2015 26929415 PMC9487661

[B116] Wang C. Horby P. W. Hayden F. G. Gao G. F. (2020). A Novel Coronavirus Outbreak of Global Health Concern. Lancet 395 (10223), 470–473. 10.1016/s0140-6736(20)30185-9 31986257 PMC7135038

[B117] Wang M. Mao C. Ouyang L. Liu Y. Lai W. Liu N. (2019). Long Noncoding RNA LINC00336 Inhibits Ferroptosis in Lung Cancer by Functioning as a Competing Endogenous RNA. Cell. Death Differ. 26 (11), 2329–2343. 10.1038/s41418-019-0304-y 30787392 PMC6889193

[B118] Wang X. Chen Y. Wang X. Tian H. Wang Y. Jin J. (2021). Stem Cell Factor SOX2 Confers Ferroptosis Resistance in Lung Cancer via Upregulation of SLC7A11. Cancer Res. 81 (20), 5217–5229. 10.1158/0008-5472.can-21-0567 34385181 PMC8530936

[B119] Wang X. Zhang C. Zou N. Chen Q. Wang C. Zhou X. (2022). Lipocalin-2 Silencing Suppresses Inflammation and Oxidative Stress of Acute Respiratory Distress Syndrome by Ferroptosis via Inhibition of MAPK/ERK Pathway in Neonatal Mice. Bioengineered 13 (1), 508–520. 10.1080/21655979.2021.2009970 34969358 PMC8805876

[B120] Wang Y. Dong Z. Zhang Z. Wang Y. Yang K. Li X. (2022). Postconditioning with Irisin Attenuates Lung Ischemia/Reperfusion Injury by Suppressing Ferroptosis via Induction of the Nrf2/HO-1 Signal Axis. Oxid. Med. Cell. Longev. 2022, 9911167. 10.1155/2022/9911167 35281462 PMC8906956

[B121] Wang Y. Tang M., PM2.5 Induces Ferroptosis in Human Endothelial Cells through Iron Overload and Redox Imbalance *.* Environ. Pollut., 2019. 254(Pt A): p. 112937. 10.1016/j.envpol.2019.07.105 31401526

[B122] Wei D. Ke Y. Q. Duan P. Zhou L. Wang C. Y. Cao P. (2021). MicroRNA-302a-3p Induces Ferroptosis of Non-small Cell Lung Cancer Cells via Targeting Ferroportin. Free Radic. Res. 55 (7), 821–830. 10.1080/10715762.2021.1947503 34181495

[B123] Wenzel S. E. Tyurina Y. Y. Zhao J. St. Croix C. M. Dar H. H. Mao G. (2017). PEBP1 Wardens Ferroptosis by Enabling Lipoxygenase Generation of Lipid Death Signals. Cell. 171 (3), 628–641.e26. 10.1016/j.cell.2017.09.044 29053969 PMC5683852

[B124] Wesselius L. J. Nelson M. E. Skikne B. S. (1994). Increased Release of Ferritin and Iron by Iron-Loaded Alveolar Macrophages in Cigarette Smokers. Am. J. Respir. Crit. Care Med. 150 (3), 690–695. 10.1164/ajrccm.150.3.8087339 8087339

[B125] Wong C.-M. Preston I. R. Hill N. S. Suzuki Y. J. (2012). Iron Chelation Inhibits the Development of Pulmonary Vascular Remodeling. Free Radic. Biol. Med. 53 (9), 1738–1747. 10.1016/j.freeradbiomed.2012.08.576 22974762 PMC3472156

[B126] Wu C.-Y. Yang Y.-H. Lin Y.-S. Chang G.-H. Tsai M.-S. Hsu C.-M. (2021). Dihydroisotanshinone I Induced Ferroptosis and Apoptosis of Lung Cancer Cells. Biomed. Pharmacother. 139, 111585. 10.1016/j.biopha.2021.111585 33862493

[B127] Wu Y. Chen H. Xuan N. Zhou L. Wu Y. Zhu C. (2020). Induction of Ferroptosis-like Cell Death of Eosinophils Exerts Synergistic Effects with Glucocorticoids in Allergic Airway Inflammation. Thorax 75 (11), 918–927. 10.1136/thoraxjnl-2020-214764 32759385

[B128] Xie S.-S. Deng Y. Guo S.-l. Li J.-q. Zhou Y.-c. Liao J. (2022). Endothelial Cell Ferroptosis Mediates Monocrotaline-Induced Pulmonary Hypertension in Rats by Modulating NLRP3 Inflammasome Activation. Sci. Rep. 12 (1), 3056. 10.1038/s41598-022-06848-7 35197507 PMC8866506

[B129] Xu B. Wang H. Chen Z. (2021). Puerarin Inhibits Ferroptosis and Inflammation of Lung Injury Caused by Sepsis in LPS Induced Lung Epithelial Cells. Front. Pediatr. 9, 706327. 10.3389/fped.2021.706327 34422728 PMC8371381

[B130] Xu R. Wu J. Luo Y. Wang Y. Tian J. Teng W. (2022). Sanguinarine Represses the Growth and Metastasis of Non-small Cell Lung Cancer by Facilitating Ferroptosis. Curr. Pharm. Des. 28 (9), 760–768. 10.2174/1381612828666220217124542 35176976

[B131] Xu W. Deng H. Hu S. Zhang Y. Zheng L. Liu M. (2021). Role of Ferroptosis in Lung Diseases. Jir Vol. 14, 2079–2090. 10.2147/jir.s307081 PMC8144020 34045882

[B132] Xu Y. Li X. Cheng Y. Yang M. Wang R. (2020). Inhibition of ACSL4 Attenuates Ferroptotic Damage after Pulmonary Ischemia‐reperfusion. FASEB J. 34 (12), 16262–16275. 10.1096/fj.202001758r 33070393

[B133] Yang N. Shang Y. (2022). Ferrostatin-1 and 3-Methyladenine Ameliorate Ferroptosis in OVA-Induced Asthma Model and in IL-13-Challenged BEAS-2B Cells. Oxid. Med. Cell. Longev. 2022, 9657933. 10.1155/2022/9657933 35154576 PMC8837457

[B134] Yang S. Ouyang J. Lu Y. Harypursat V. Chen Y. (2022). A Dual Role of Heme Oxygenase-1 in Tuberculosis. Front. Immunol. 13, 842858. 10.3389/fimmu.2022.842858 35281042 PMC8913507

[B135] Yang W. S. SriRamaratnam R. Welsch M. E. Shimada K. Skouta R. Viswanathan V. S. (2014). Regulation of Ferroptotic Cancer Cell Death by GPX4. Cell. 156 (1-2), 317–331. 10.1016/j.cell.2013.12.010 24439385 PMC4076414

[B136] Yang W. S. Stockwell B. R. (2016). Ferroptosis: Death by Lipid Peroxidation. Trends Cell. Biol. 26 (3), 165–176. 10.1016/j.tcb.2015.10.014 26653790 PMC4764384

[B137] Yang Y. Tai W. Lu N. Li T. Liu Y. Wu W. (2020). lncRNA ZFAS1 Promotes Lung Fibroblast-To-Myofibroblast Transition and Ferroptosis via Functioning as a ceRNA through miR-150-5p/SLC38A1 axis. Aging 12 (10), 9085–9102. 10.18632/aging.103176 32453709 PMC7288977

[B138] Ye L. F. Chaudhary K. R. Zandkarimi F. Harken A. D. Kinslow C. J. Upadhyayula P. S. (2020). Radiation-Induced Lipid Peroxidation Triggers Ferroptosis and Synergizes with Ferroptosis Inducers. ACS Chem. Biol. 15 (2), 469–484. 10.1021/acschembio.9b00939 31899616 PMC7180072

[B139] Yoshida M. Minagawa S. Araya J. Sakamoto T. Hara H. Tsubouchi K. (2019). Involvement of Cigarette Smoke-Induced Epithelial Cell Ferroptosis in COPD Pathogenesis. Nat. Commun. 10 (1), 3145. 10.1038/s41467-019-10991-7 31316058 PMC6637122

[B140] Yu S. Jia J. Zheng J. Zhou Y. Jia D. Wang J. (2021). Recent Progress of Ferroptosis in Lung Diseases. Front. Cell. Dev. Biol. 9, 789517. 10.3389/fcell.2021.789517 34869391 PMC8635032

[B141] Zhang F. Liu H. Liu H. (2021). Identification of Ferroptosis-Associated Genes Exhibiting Altered Expression in Pulmonary Arterial Hypertension. Mbe 18 (6), 7619–7630. 10.3934/mbe.2021377 34814266

[B142] Zhang J. Liu Y. Guo Y. Zhao Q. (2020). GPX8 Promotes Migration and Invasion by Regulating Epithelial Characteristics in Non‐small Cell Lung Cancer. Thorac. Cancer 11 (11), 3299–3308. 10.1111/1759-7714.13671 32975378 PMC7606007

[B143] Zhang X. Sui S. Wang L. Li H. Zhang L. Xu S. (2020). Inhibition of Tumor Propellant Glutathione Peroxidase 4 Induces Ferroptosis in Cancer Cells and Enhances Anticancer Effect of Cisplatin. J. Cell. Physiol. 235 (4), 3425–3437. 10.1002/jcp.29232 31556117 PMC13482695

[B144] Zhang Y. Zheng L. Deng H. Feng D. Hu S. Zhu L. (2022). Electroacupuncture Alleviates LPS-Induced ARDS through α7 Nicotinic Acetylcholine Receptor-Mediated Inhibition of Ferroptosis. Front. Immunol. 13, 832432. 10.3389/fimmu.2022.832432 35222419 PMC8866566

[B145] Zhao J. Dar H. H. Deng Y. St. Croix C. M. Li Z. Minami Y. (2020). PEBP1 Acts as a Rheostat between Prosurvival Autophagy and Ferroptotic Death in Asthmatic Epithelial Cells. Proc. Natl. Acad. Sci. U.S.A. 117 (25), 14376–14385. 10.1073/pnas.1921618117 32513718 PMC7321965

[B146] Zhao J. Maskrey B. Balzar S. Chibana K. Mustovich A. Hu H. (2009). Interleukin-13-induced MUC5AC Is Regulated by 15-lipoxygenase 1 Pathway in Human Bronchial Epithelial Cells. Am. J. Respir. Crit. Care Med. 179 (9), 782–790. 10.1164/rccm.200811-1744oc 19218191 PMC2675565

[B147] Zhao J. O'Donnell V. B. Balzar S. St. Croix C. M. Trudeau J. B. Wenzel S. E. (2011). 15-Lipoxygenase 1 Interacts with Phosphatidylethanolamine-Binding Protein to Regulate MAPK Signaling in Human Airway Epithelial Cells. Proc. Natl. Acad. Sci. U.S.A. 108 (34), 14246–14251. 10.1073/pnas.1018075108 21831839 PMC3161579

[B148] Zhao K. Huang J. Dai D. Feng Y. Liu L. Nie S. (2020). Serum Iron Level as a Potential Predictor of Coronavirus Disease 2019 Severity and Mortality: A Retrospective Study. Open Forum Infect. Dis. 7 (7), ofaa250. 10.1093/ofid/ofaa250 32661499 PMC7337740

[B149] Zheng J. Conrad M. (2020). The Metabolic Underpinnings of Ferroptosis. Cell. Metab. 32 (6), 920–937. 10.1016/j.cmet.2020.10.011 33217331

[B150] Zhou F. Yu T. Du R. Fan G. Liu Y. Liu Z. (2020). Clinical Course and Risk Factors for Mortality of Adult Inpatients with COVID-19 in Wuhan, China: a Retrospective Cohort Study. Lancet 395 (10229), 1054–1062. 10.1016/s0140-6736(20)30566-3 32171076 PMC7270627

[B151] Zhou H. Li F. Niu J. Y. Zhong W. Y. Tang M. Y. Lin D. (2019). Ferroptosis Was Involved in the Oleic Acid-Induced Acute Lung Injury in Mice. Sheng Li Xue Bao 71 (5), 689–697. 31646322

[B152] Zou H. X. Qiu B. Q. Lai S. Q. Zhou X. L. Gong C. W. Wang L. J. (2021). Iron Metabolism and Idiopathic Pulmonary Arterial Hypertension: New Insights from Bioinformatic Analysis. Biomed. Res. Int. 2021, 5669412. 10.1155/2021/5669412 34722766 PMC8556088

